# Transcriptome Profiling of *Giardia intestinalis* Using Strand-specific RNA-Seq

**DOI:** 10.1371/journal.pcbi.1003000

**Published:** 2013-03-28

**Authors:** Oscar Franzén, Jon Jerlström-Hultqvist, Elin Einarsson, Johan Ankarklev, Marcela Ferella, Björn Andersson, Staffan G. Svärd

**Affiliations:** 1 Department of Cell and Molecular Biology, Karolinska Institutet, Stockholm, Sweden; 2 Department of Cell and Molecular Biology, BMC, Uppsala University, Uppsala, Sweden; National University of Singapore, Singapore

## Abstract

*Giardia intestinalis* is a common cause of diarrheal disease and it consists of eight genetically distinct genotypes or assemblages (A-H). Only assemblages A and B infect humans and are suggested to represent two different *Giardia* species. Correlations exist between assemblage type and host-specificity and to some extent symptoms. Phenotypical differences have been documented between assemblages and genome sequences are available for A, B and E. We have characterized and compared the polyadenylated transcriptomes of assemblages A, B and E. Four genetically different isolates were studied (WB (AI), AS175 (AII), P15 (E) and GS (B)) using paired-end, strand-specific RNA-seq. Most of the genome was transcribed in trophozoites grown *in vitro*, but at vastly different levels. RNA-seq confirmed many of the present annotations and refined the current genome annotation. Gene expression divergence was found to recapitulate the known phylogeny, and uncovered lineage-specific differences in expression. Polyadenylation sites were mapped for over 70% of the genes and revealed many examples of conserved and unexpectedly long 3′ UTRs. 28 open reading frames were found in a non-transcribed gene cluster on chromosome 5 of the WB isolate. Analysis of allele-specific expression revealed a correlation between allele-dosage and allele expression in the GS isolate. Previously reported *cis*-splicing events were confirmed and global mapping of *cis*-splicing identified only one novel intron. These observations can possibly explain differences in host-preference and symptoms, and it will be the basis for further studies of *Giardia* pathogenesis and biology.

## Author Summary

*Giardia* is a single cell intestinal parasite and a common cause of diarrhea in humans and animals. *Giardia* is an unusual eukaryote by possessing two nuclei, a highly reduced genome and simple transcriptional apparatus. We have characterized the transcriptome of *Giardia* at single nucleotide resolution, which allowed the calculation of digital gene expression values for the complete set of genes. We performed a comparison of gene expression divergence across three genotypes. Most of the genes were transcribed, and the data were used to refine and correct gene models. Several gene expression differences were identified between the genotypes. A non-transcribed cluster of genes was detected on chromosome 5, likely representing a silenced region. The data also allowed mapping of transcript termini, which provided the first global view of 3′ untranslated regions in this parasite. This study also gives the first genome-wide evidence of transcription of allelic variants in *Giardia*. In this study, we provide novel insights into the transcriptome of an important human pathogen and model eukaryote. The findings reported here likely relate to the lifestyle of this parasite and its adaptation to parasitism. The data provide starting points for functional investigation of *Giardia's* biology and diplomonads generally.

## Introduction

The diplomonad *Giardia intestinalis* (syn. *G. lamblia*, *G. duodenalis*) is a parasitic protozoan and common cause of diarrhea in humans and animals [1]. Individuals become infected with *G. intestinalis* upon ingestion of contaminated water or food. Giardiasis contributes to malnutrition and malabsorption in developing countries, leading to growth retardation and failure of children to thrive. Besides being a medically important pathogen, *G. intestinalis* has several unusual characteristics, including the presence of two nuclei in trophozoites, a mitochondria-derived organelle known as the mitosome and a highly reduced genome. The life cycle of *G. intestinalis* is relatively simple, consisting of the environmentally stable and infectious cyst and the flagellated trophozoite [1]. The cyst is the dormant stage of the parasite and contains four tetraploid nuclei; i.e., sixteen copies of the genome per cyst. The trophozoite is the replicative stage, and it is binucleated, i.e. containing four to eight copies of the genome.

*G. intestinalis* is currently being partitioned into eight genotypes, also known as assemblages (A to H), displaying varying levels of host specificity. Only assemblages A and B are associated with human infections, but also infect a broader range of mammals and may have potential for zoonotic transmission [2], [3]. Parasites of assemblage E are predominantly associated with hoofed animals. The *G. intestinalis* genome is spread in five chromosomes, and the haploid genome size is ∼12 Mb. Genome sequencing of representative isolates of assemblages A, B, and E has been performed [4]–[6]. The three representative isolates WB, GS, and P15 belong to assemblages A, B, and E, and comparative genomics showed an average sequence identity of ∼77% between assemblage A and B, and ∼87% between A and E. With few exceptions, genes lack introns, and ∼86% of the genome is protein-coding. Each of the three genomes contains ∼5000 genes, but the precise number of annotated genes differs slightly because of differences in genome finishing. Relatively few assemblage-specific genes have been identified and most genomic differences are found in intergenic regions and in the *Giardia*-specific gene families [5], [6].

The mechanisms underlying transcriptional regulation in *G. intestinalis* are poorly understood. Genome analyses have indicated short A+T-rich promoters, which seem to be a prerequisite for recognition by the transcriptional machinery [7]–[9]. Known regulatory promoter elements are only reported for a few developmentally regulated genes [8], [10]. The RNA processing machinery of *G. intestinalis* is simplified compared to well-studied eukaryotes [4], [11]–[13], which has raised questions whether it resulted from adaptation to a parasitic lifestyle or is a characteristic of the ancestral eukaryote. 21 of 28 of the eukaryotic RNA polymerase II polypeptides are encoded in the genome, but only 4 of the 12 general transcription initiation factors [13]. Moreover, a highly diverged gene encoding a TATA-binding protein is present and TFIIB is completely missing. Compared to other eukaryotes, *G. intestinalis* lacks some components of the exosome complex, and the mechanism responsible for nonsense-mediated mRNA decay appears not to be fully present [14], [15]. *cis*- and *trans*-splicing appear to be very limited, with only a few documented examples [16]–[20]. The global occurrence of splicing in *G. intestinalis* remains unknown. While the RNA interference machinery is present and operational at some extent [4], [21], [22], it is not completely analogous with metazoan RNAi. Small, non-coding RNAs have been documented [22], [23] and are implicated in gene regulation [23] and antigenic variation [21].

Previous studies of transcription in *G. intestinalis* have employed Serial Analysis of Gene Expression (SAGE) [24] and oligonucleotide arrays [25]–[29]. SAGE was applied to characterize ten stages of the life cycle, and uncovered a certain extent of differential gene expression throughout the life cycle [24]. SAGE also indicated abundant antisense transcription. Promoters in *G. intestinalis* are suggested to be bidirectional, in part explaining the production of antisense transcripts [30]. In addition, promiscuous transcription driven by ‘cryptic’ promoters, containing simple A+T-rich stretches also contributes to antisense transcription [30]. However, transcript discovery using SAGE is limited by under-representation of genes with low expression levels, and this method has largely been replaced by high-throughput sequencing assays. Array-based techniques have been used extensively [25]–[29], but these techniques suffer from template cross-hybridization, low dynamic range, and inability to identify transcriptional start/stop sites.

In the present study we describe transcriptome sequencing (RNA-seq) of four *G. intestinalis* isolates from three assemblages (2 of A, 1 of B, and 1 of E). Our aims were to give a more comprehensive overview of the *G. intestinalis* transcriptome and to perform an evolutionary comparison of gene expression. We also describe the first global mapping and analysis of polyadenylation sites in *G. intestinalis*. We found that most of the *G. intestinalis* genome was transcribed in trophozoites grown *in vitro*, albeit at vastly different levels. This is the first study to carry out deep gene expression profiling of diverged *G. intestinalis* isolates, and it provides new insights into the transcriptional landscape of an important human pathogen and model eukaryote.

## Materials and Methods

### Accession Numbers and Genome Sequences

The RNA-seq data discussed in this publication have been deposited in NCBI's Gene Expression Omnibus [31] and are accessible through GEO Series accession number GSE36490 (http://www.ncbi.nlm.nih.gov/geo/query/acc.cgi?acc=GSE36490). The genome sequence of AS175 has been deposited in the European Nucleotide Archive under the accession number CAHQ00000000. Genome sequences and annotations of WB, P15, and GS were downloaded from GiardiaDB (v.2.3; GenBank accession numbers: AACB02000000, ACVC01000000, and ACGJ01000000) [32]. Gene coordinates used in the present study can be found in Table S1.

### Sample Preparation and RNA Isolation

WB and GS are clones originally isolated from human infections [33], [34] and are sub-grouped into assemblages A(I) and B(IV), respectively. WB and GS are well-characterized in the literature and have been genome sequenced [4], [5]. AS175 was recently recovered from a human individual and belongs to assemblage A(II). We studied gene expression of AS175 after 4 and 33 passages *in vitro*. The P15 clone belongs to assemblage E (a livestock clade) and it was originally isolated from a symptomatic pig [35]. Trophozoites of WB, GS, and P15 were grown in TYDK and prepared as in [6], and trophozoites of AS175 were prepared as described in [36]. AS175 trophozoites were grown in slanted culture tubes to pre-confluence and harvested by ice-slush incubation followed by centrifugation at 4°C for 5 min. The supernatant was removed by aspiration, and the pellets were dissolved in TRIzol reagent (Invitrogen, 15596-026) and stored at −20°C for less than one week before extraction. WB, GS, and P15 cells were cultured at 37°C to 60–70% confluence in 25 cm^2^ cell culture flasks containing 60 ml TYDK. Cells were harvested by cell scraper, transferred to 50 ml Falcon tubes and then pelleted by centrifugation at 2500 rpm at 4°C for 5 min. The medium was removed by vacuum suction, and the pellets were brought up in at least 10 pellet volumes of ice-cold PBS. The pooled pellets were distributed evenly to 1.5 ml centrifuge tube and pelleted at 13,000 rpm at 4°C for 1 min. The supernatant was removed, and cells corresponding to 2–3 flasks were dissolved in 1 ml TRIzol reagent. The TRIzol dissolved material was stored at −20°C for less than one week before extraction. Thawed tubes were incubated at room temperature for 5 min followed by addition of chloroform (0.2 ml/ml TRIzol). The tubes were shaken vigorously and incubated for 3 min a room temperature before centrifugation at 12,000×g for 15 min at 4°C. The water phase containing the RNA was collected and extracted with 600 µl chloroform. The tube was vigorously shaken and centrifuged at 15,000 rpm for 15 min at 4°C. The water-phase was collected and precipitated by addition of 1.1 ml ice-cold 100% EtOH and 40 µl 5M NH_4_OAc. The sample was incubated at least 2 hours at −20°C before being centrifuged at 12,000×g for 30 min at 4°C. The pellets were washed with addition of 1 ml 75% EtOH, vortexed and centrifuged at 7500×g for 5 min at 4°C. The supernatant was removed, and the wash was repeated once. The RNA pellets were air-dried and were each dissolved in 30 µl DEPC water (Ambion). The quality and quantity were analyzed using NanoDrop, BioAnalyzer and agarose gel electrophoresis.

### Selection of PolyA Transcripts

PolyA selection was performed using the Poly(A) purist MAG system (Ambion, AM1922). The RNA (120 µl) from the previous step was diluted to 200 µl with DEPC water and precipitated using 3 volumes of ice-cold 100% EtOH and 20 µl 3M Sodium acetate, pH 5.5. The samples were incubated at −70°C for 2 hours and centrifuged at 15,000 rpm for 30 min at 4°C. The RNA pellet was washed with 75% EtOH and re-suspended in 60 µl DEPC water. 100 µg of total RNA respectively from WB, GS, and P15 was subjected to polyA selection according to the manufacturer's instructions. The two AS175 samples each contained less than 15 µg of total RNA and were processed according to the protocol for 30 µg or less of starting material. The purified material was pooled and precipitated by addition of 1.1 ml ice-cold 100% EtOH, 40 µl 5M NH_4_OAc and 1 µl of glycogen (5 mg/ml). The RNA was precipitated at −70°C for 3 hours and centrifuged at 15,000 rpm for 30 min at 4°C. The pellet was washed once with 75% EtOH, air-dried and dissolved in 20 µl Nuclease-free water (Ambion). Quality and quantity of the RNA were evaluated using NanoDrop and BioAnalyzer.

### Removal of DNA Contaminants

The purified mRNA from WB, GS, and P15 was treated with the Turbo DNA-free kit (Applied Biosystems, AM1907). The total RNA of AS175 was DNase treated before polyA selection, using no more than 10 µg in a single reaction. Eluates from mRNA selection or total RNA were mixed with 5 µl Turbo DNase buffer (10×), 1 µl Turbo DNase in a total volume of 50 µl. The samples were incubated in a thermo-cycler for 30 min at 37°C. The samples were each treated with 5 µl of DNase Inactivation reagent for 5 min with occasional agitation. The beads were pelleted, and the supernatant aspirated. 45 µl of the recovered supernatant was mixed with 4.5 µl 5M NH_4_OAc and 1 µl of glycogen (5 mg/ml) and 112.5 µl 100% EtOH. The samples were incubated at −70°C over-night. The samples were centrifuged at 15,000 rpm for 30 min at 4°C. The pellets were washed with 75% EtOH, air-dried and dissolved in 10 µl of Nuclease-free water (Ambion). The quality and quantity of the RNA was evaluated using NanoDrop and BioAnalyzer.

### Library Preparation and Sequencing

Strand-specific RNA sequencing libraries, compatible with the Illumina sequencing chemistry, were prepared using the ScriptSeq mRNA-Seq library Preparation Kit (SS10906, Epicentre) according to the manufacturer's protocol (Lit. #313) with a few modifications. The amounts of mRNA used in library construction were the following: WB, 200 ng; GS, 500 ng; P15, 450 ng; AS175_P4_, 75 ng; and AS175_P33_, 350 ng. During RNA fragmentation, samples were incubated at 80°C for 5 min in a thermo-cycler. In step D, purification of the di-tagged cDNA was performed with the MinElute PCR purification kit (Qiagen, 28006) according to the manufacturer's instructions. The di-tagged cDNA was eluted by addition of 20 µl of EB buffer. ScriptSeq Index PCR Primers (1–12; Epicentre, RSBC10948) were employed instead of the reverse primer in the kit. The following barcodes in the indexing primers were used for the ScriptSeq libraries: WB, Barcode 3 (GCCTAA); P15, Barcode 2 (ACATCG); GS, Barcode 4 (TGGTCA); AS175_P4_, Barcode 5 (CACTGT); and AS175_P33_, Barcode 6 (ATTGGC). The barcodes were added employing the FailSafe PCR Enzyme mix (Epicentre, FSE51100) for 10 cycles for the WB, GS, and P15 samples, whereas 12 cycles were employed for the AS175_P4_ and AS175_P33_ samples. The libraries were purified using the MinElute PCR purification kit as described previously and eluted in 10 µl EB buffer. The libraries were quantified using NanoDrop and BioAnalyzer. The quality of the libraries was evaluated by TOPO-TA cloning of fragments and sequencing using dye-terminator sequencing as described in [37]. An 8 pM solution of the sequencing libraries in equimolar amounts was subjected to cluster generation on the cBot instrument (Illumina Inc.). Libraries were sequenced as 2×100-nt paired-end reads on an Illumina HiSeq 2000 instrument at the SNP&SEQ Technology Platform at the Science for Life Laboratory (Uppsala University). The libraries were sequenced independently on two different lanes to assess technical variation. Base calling was done on the instrument (RTA 1.10.36) and the resulting .bcl files were converted to qseq format with tools provided by OLB-1.9.0 (Illumina Inc.). Fastq sequence files were generated using CASAVA 1.7.0 (Illumina Inc.). Additional statistics on sequence quality were compiled from the base call files with an in-house script.

### Scripts and Statistics

Scripts were written in Perl v.5.12.4 and R v.2.14.0 and are available on request. Pearson's correlation coefficient *r* was used to compare distributions. In multiple hypothesis testing, *p*-values were corrected using the Benjamini-Hochberg procedure. The following external R-libraries were used in addition to the standard: ggplot2, IDPmisc (the Image function), gplots (the heatmap.2 function), and ade4 (the mantel.rtest function).

### Defining Orthologs

Orthologs were identified at the protein-level using a best reciprocal BLAST heuristic implemented in the program Proteinortho v.4.25 (settings: -e = 1e-10, -p = blastp, -id = 25, -cov = 0.5, -conn = 0.1, -m = 0.95) [38]. Orthologs were required to be in single copy and full-length, i.e. not split on two or more contigs.

### Sequence Data Mapping

Data of the same library but from different sequencing lanes were merged. The data were then searched for read-pairs containing the library adapter. Adapter sequences were identified in 45 to 61% of the read-pairs, indicating sequencing of DNA fragments <200 nt. Before the data were mapped, adapter-containing read-pairs were identified and the overlap of left and right reads was used to join the mates into one sequence. The program SeqPrep (downloaded May 21, 2012; https://github.com/jstjohn/SeqPrep) was used to join these reads (non-default parameter: −6). Joined reads were then mapped as single-end reads to the genomes. Moreover, read-pairs without the adapter were mapped as paired-end reads, since these read-pairs resulted from sequencing of DNA fragments >200 nt. All reads were aligned with bowtie v.2.0.0-beta6 [39]. Reads reporting more than one match were kept pseudo-randomly using bowtie's default options.

### Calculating Digital Gene Expression Values

Transcript levels were calculated from the mapped sequences as fragments per kilobase per million fragments mapped (FPKM), using cufflinks v.2.0.0 [40]. The setting ‘–library-type fr-secondstrand’ was used. The threshold for transcription was set to 0.5 FPKM; i.e., genes with FPKM≤0.5 were not regarded expressed.

### Reverse Transcription and Quantitative PCR Analysis

Total RNA from four 10 ml cultures of *G. intestinalis* WB trophozoites was prepared using the TRIzol reagent as specified by the manufacturer (Ambion, Life Technologies). RNA samples were pooled and treated with DNAse-I (Fermentas). The quality and quantity of the RNA preparation were assessed with TBE-agarose gel and Nanodrop. Polyadenylated cDNA was prepared using the Thermo Scientific RevertAid H Minus First Strand cDNA Synthesis Kit using 1.5 to 2.0 µg of DNAse-I treated total RNA per reaction. Minus template and minus Reverse transcriptase controls were assessed. Expression levels of 49 transcripts of WB were confirmed by real time PCR using the Thermo Scientific Maxima SYBR Green/ROX qPCR master mix. Each reaction was performed in triplicates using 0.5 µl of cDNA. Two primer sets were designed and used for genes with length above 2.5 kb.

### Estimating Technical and Biological Variation

Technical replicates were from the same biological sample and were split after library preparation. Data from each sequencing lane were aligned, and FPKM counts from lanes 1 and 2 were then compared. Biological variation was estimated using the AS175 samples. Differentially expressed genes were identified using a χ^2^ test of the fold change compared with fold changes obtained from technical replicates.

### Microarray and SAGE Comparisons

Raw microarray data were provided by the A. Hehl lab and processed as described in [29], relying on the R-libraries limma and multtest. Oligonucleotide probes were aligned with the WB genome using bowtie v.2.0.0-beta6 [39] (default settings). Probes were assigned to genes if completely contained in open reading frames. Genes shorter than 500 bp were not considered. Curated SAGE data were downloaded from GiardiaDB [32].

### Comparison of Gene Expression and Genetic Distances

Orthologs were aligned with ClustalW v.2.1 and genetic distances were computed with TREE-PUZZLE v.5.2 [41]. Euclidean distances were computed using the ‘dist’ function in R. Mantel's test was used to compare matrices as implemented in the mantel.rtest function (ade4 package).

### Feature Prediction and Gene Ontology

Signal peptides, and *trans*-membrane domains were predicted with SignalP v.4.0 [42] and TMHMM v.2.0c [43], respectively. The program map2slim was used to map Gene Ontology [44] annotations to broader groups. Gene Ontology annotations were downloaded from GiardiaDB. The generic slimmed Gene Ontology was employed. DNA motifs were searched for using MEME v.4.8.1 (‘-mod’ set to zoop or anr, ‘nmotifs’ set to 6, and the motif length 6 to 8) [45].

### Mapping Transcript 3′ Ends

PolyA reads (referred to as polyA tags) containing at least 7 adenines were extracted from the left mate of read-pairs. The polyA stretch was then removed. Subsequently, we searched and removed the adapter using cutadapt v.0.9.5 (setting: -q 20; http://code.google.com/p/cutadapt/). PolyA tags were then mapped using bowtie v.2.0.0-beta6 (default settings). Alignments with mapping quality ≤40 were removed. PolyA sites were clustered if located within 10 nt from each other. Only clusters containing ≥4 polyA tags were kept for subsequent analyses. Clusters were then assigned to ORFs based on proximity. The most frequent polyA site of each cluster was selected as the 3′ end.

### 3′-Rapid Amplification of cDNA Ends (3′ RACE)

Polyadenylation sequences were experimentally investigated using the ExactSTART Eukaryotic mRNA 5′- & 3′-RACE Kit (Epicentre, ES80910). The guidelines and recommendations of the manufacturer were followed. Total RNA from WB was isolated using the TRIzol extraction process according to the manufacturer's protocol (Lit. # 293). Total RNA (10 µg) was treated with APex phosphatase and extracted by phenol/chloroform (1∶1) and chloroform∶isoamyl alcohol (24∶1) followed by concentration with isopropanol precipitation. The sample was treated by Tobacco acid pyrophosphatase and then the 3′RACE oligo was ligated by T4 RNA ligase to RNAs carrying a 3′ monophosphate. First-strand cDNA was synthesized by MMLV Reverse Transcriptase and the cDNA synthesis primer that includes an oligod(T) with a primer sequence for downstream PCR amplification. Second-strand cDNA synthesis and amplification were performed by adding PCR Primer 1 and PCR Primer 2 followed by PCR amplification using FailSafe PCR Premix E and FailSafe PCR enzyme mix (Epicentre, FSE51100). The cDNA was diluted 1∶100, PCR primer 2 and locus specific forward primers were employed. The samples were amplified for 21 cycles with cycling parameters as recommended. Locus specific 3′ RACE reactions were prepared by combining 1 µl cDNA, 21 µl nuclease-free water, 1 µl PCR primer 1, 0.75 µl 20 µM locus specific reverse primer, and 25 µl FailSafe premix E. The cycler was paused at 95°C and 1 µl of FailSafe PCR enzyme was added. PCR was then performed by 95°C for 30 seconds (initial denaturation) followed by 35 cycles of: 94°C for 20 s, 60°C for 20 s, 72°C for 20 s. The PCR reactions were analyzed by agarose gel electrophoresis and the remainder were purified by the MinElute PCR purification kit and eluted in 10 µl EB. The eluates from 3′ RACE reactions were cloned by TOPO-TA cloning and were sequenced using dye-terminator sequencing as described in [37].

### Polyadenylation Signals

Polyadenylation signals were identified as described in [46]. 3′ fragments of 40 nucleotides length (position −40 to −1 relative to the polyA site) were extracted. The search was conducted for hexamers, with the scattering threshold set to 6 (empirically determined). For each such 3′ fragment, all possible hexamer combinations were extracted and the most common hexamer was saved. Subsequently, all 3′ fragments containing this hexamer were removed from the database before searching for the next most frequent hexamer. The distribution of hexamers was also considered. For each motif, the average position and standard deviation were calculated.

### Allele-specific Expression

Heterozygotic loci were identified using genomic Roche 454 reads from [5]. Roche 454 reads were aligned with GS contigs ≥10 kb using the program bwa v.0.6.1 bwasw [47]. 782,180 reads of the median length 239 nt mapped uniquely to the contigs (median coverage = 14X). Alignments were used to identify mismatches, corresponding to heterozygous loci of the tetraploid genome. Loci containing sequence reads with alignment gaps at the heterozygous position were not considered. Only sites with 10 to 80X coverage were scanned. The coverage constraints were arbitrarily selected to avoid low quality regions and putatively collapsed repeats, and resulted in 8,755,898 sites that fell within the defined interval. In theory, it is possible to find heterozygous loci containing four different nucleotide variants (one variant from each of the four haplotypes), but most heterozygous loci contain only two nucleotide variants [5]. The heterozygosity had to be supported by ≥4 reads and located >250 bp from contig ends.

Simulated reads were generated using the program pIRS v.1.1.0 [48] with the settings ‘-a 0 -m 100 -l 100 -x 100 -v 10’. Simulated reads were aligned with the masked genome using bowtie2 with the setting ‘--very-sensitive’. RNA-seq data of GS were re-aligned using bowtie2 with the masked GS reference using the ‘--very-sensitive’ option. Heterozygous loci with RNA-seq coverage ≥20X were analyzed. Only RNA-seq reads of the same direction as the gene was counted, i.e. ignoring antisense reads. SNP pairs of the same phase were identified from genomic reads using an in-house Perl script.

### Splice Site Mapping and PCR Validation

Splice sites were mapped with TopHat v.2.0.4 (minimum intron length of 10 nt) [49]. Potential introns predicted by TopHat were investigated by PCR amplification of genomic DNA (gDNA) and RNA (cDNA) harvested from *G. intestinalis* WB trophozoites. Primer pairs were designed on each side of the predicted intron borders to give a size difference upon a potential splicing event. Each reaction consisted of one Ready-To-Go PCR beads (GE Healthcare), 1 µl 10 µM primer-pair mix, 1 µl template (gDNA; 10 ng/µl or cDNA or -RT cDNA) and 23 µl ddH_2_O. The cDNA was prepared according to the method in the ‘Reverse Transcription and Quantitative PCR Analysis’ section. Control reactions where reverse transcriptase had been omitted from cDNA synthesis were employed to judge the amount of gDNA remaining in the cDNA preparations. PCRs were performed with following cycling program: 95°C 5 min followed by 32 cycles of 95°C 5 min, 58°C 30 s, 72°C 1 min/kb of amplicon size, terminating with 10 min at 72°C. The PCR products were separated by agarose gel electrophoresis and stained with ethidium bromide. GeneRuler 1 kb and GeneRuler 100 bp ladders (Fermentas) were used to size the PCR products. Relevant bands were gel purified using the QIAquick Gel Extraction kit (QIAGEN) and sequenced using amplicon specific forward and reverse primers.

## Results/Discussion

### Genome-wide Analysis of Transcription by Paired-end RNA-Seq

**Figure 1:**
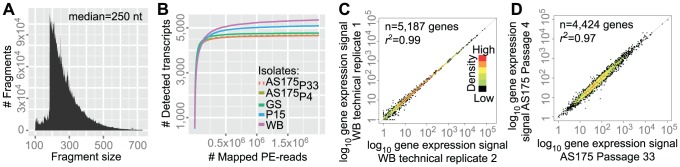
RNA-seq technical details. (A) Insert size histogram of sequenced cDNA fragments inferred from mapped paired-end reads. The plotted data are from the WB isolate. The x- and y-axes show the fragment size in nucleotides and the frequency, respectively. The median length was 250 nt. (B) The relationship between detected transcripts and mapped paired-end reads. The x- and y-axes show the number of mapped reads and the number of detected transcripts, respectively. Colors correspond to: violet (WB), blue (P15), green (GS), yellow (AS175_P4_), and dotted (AS175_P33_). The plateau indicates saturation (deeper sequencing do not lead to detection of new transcripts). Since the reference genomes slightly differ in finishing, the plateau y-values are different. (C) Gene expression correlation of technical replicates (WB isolate). Technical replicates 1 and 2 are from the same sequencing library (biological sample) but sequenced independently on different lanes. Dots represent genes. The x- and y-axes show log_10_-scaled FPKM of technical replicates 1 and 2 respectively (values were incremented by 1 before transformation). The blue line corresponds to equal expression. Colors represent overlap in the plot; i.e., black means a single gene and red means higher plotting density. (D) Correlation of gene expression between *in vitro* passages 4 and 33 of the AS175 isolate, i.e., correlation of biological replicates.

To study gene expression in *Giardia intestinalis* we selected four different isolates representing three assemblages (syn. genotypes): WB, AS175, P15, and GS. Total polyadenylated RNA was converted to double-stranded cDNA, the cDNA was fragmented, and sequencing libraries were prepared according to a strand-specific protocol (Materials and Methods). The libraries were sequenced as 2×100-nt paired-end reads on an Illumina HiSeq 2000 instrument. Each library was sequenced on two lanes to give an estimate of technical variation. Sequencing generated 33 to 41 million 100-nt read-pairs per library. 92 to 95% of the total data aligned with the reference genomes (Table 1). The median DNA fragment length was 250 nt (WB; Figure 1A; Figure S1). Four *G. intestinalis* genome sequences were used for mapping the RNA-seq data, and the mapped data were then used to calculate digital gene expression levels, formulated as fragments per kilobase per million fragments mapped (FPKM) [50]. Technical variation was estimated by comparing data from different sequencing lanes, which indicated very low technical variation (*r* ^2^ = 0.99; Figure 1C; Figure S2).

**Table 1:** Raw and mapped sequence data.

	Isolate/Metric	WB (A)	AS175_P4_ (A)	AS175_P33_ (A)	P15 (E)	GS (B)
**Original raw data** a	**# read-pairs**	41,172,817	33,637,791	38,914,235	38,278,044	33,307,482
**Short fragments** b	**# read-pairs**	25,067,158	16,414,699	19,730,224	22,382,992	15,262,410
	**Avg. len. after merging (nt)** d	108	114	111	107	114
	**∑ nt** e	2,702,404,625	1,876,878,086	2,194,344,812	2,399,592,422	1,746,481,426
	**# mapped**	24,109,674	15,881,238	19,134,117	22,060,153	14,799,395
**Long fragments** c	**# read-pairs**	15,483,650	16,890,474	18,761,168	15,346,782	17,818,939
	**# properly mapped read-pairs**	12,781,862	15,160,384	16,914,770	13,444,794	16,473,686
	**# read-pairs where left or right mapped**	996,886	788,959	829,952	932,191	596,725
**Total**	**Total % mapped**	92% (37,888,422/41,172,817)	94% (31,830,581/33,637,791)	94% (36,878,839/38,914,235)	95% (36,437,138/38,278,044)	95% (31,869,806/33,307,482)

a Number of 2×100-nt read-pairs resulting from HiSeq 2000 sequencing.

b Read-pairs containing the sequencing adapter. The left and right reads overlap due to sequencing of DNA fragments <200-nt. These reads (left and right) were merged and mapped as single-end reads.

c Reads without the adapter sequence. These resulted from sequencing of DNA fragments >200-nt. The left and right mate did not overlap and these were mapped as pairs.

d The average sequence length after merging left and right reads.

e The combined number of nucleotides after merging of left and right reads but before mapping.

The number of mapped reads was compared with the number of detected transcripts (Figure 1B). The curve in Figure 1B reaches the plateau at around 0.5 million mapped reads, indicating saturation of the data set. The RNA-seq coverage on open reading frames (ORFs) was assessed using the average fold coverage across all ORFs (Figure S3). The coverage was relatively uniform, but with a drop towards the 5′ end, likely related to the random hexamer-priming step [51]. The reproducibility of gene expression measurements was assessed using two samples of the isolate AS175. The AS175 samples were harvested from passages 4 and 33, and the two samples were essentially biological replicates. The AS175 samples displayed almost identical and overlapping curves (Figure 1B), indicating a strong correlation in gene expression (*r* ^2^ = 0.97, *p*<2.2e-16; Figure 1D). The biological replicates confirmed the reproducibility of the method and indicated limited environmental variance.

The genomic reference sequences used in the present study contain the complete set of coding sequences of the studied isolates, but the genomes are currently fragmented in many contigs. We identified orthologs of the four genomes using protein BLAST, which outlined 4175 clusters of 1∶1∶1∶1 (four-way) orthologs (genes that are unambiguously present in a single copy per haploid genome). Finally, since the success of RNA-seq heavily relies on the accuracy of gene models, we refined the annotation prior to RNA-seq analysis, adding 179, 187, and 385 gene models to the WB, P15 and GS genomes, respectively (Table S1).

The threshold for transcription was set to 0.5 FPKM. Transcription was identified from 93.7% (WB), 98.3% (AS175), 96.3% (P15), and 97.3% (GS) of the annotated ORFs. We excluded 388 short ORFs (length <250 bp) with exceedingly low or high FPKM values, as these are likely not encoding proteins. We found higher fractions of transcribed genes when only 1∶1∶1∶1 orthologs were considered: 99.5% (WB; 4,157/4,175), 99.8% (AS175; 4,168/4,175), 99.6% (P15; 4,160/4,175), and 99.7% (GS; 4,166/4,175). The median FPKM of the 5% highest and lowest expressed genes was 1118.76 and 1.28; i.e., an 873X fold difference (considering genes longer than 500 bp; and FPKM>0.5). The wide dynamic range illustrates the high sensitivity of RNA-seq over microarrays.

### Validation of RNA-Seq Measurements

The gene expression measurements were compared with normalized fluorescent intensities obtained from microarray experiments. The work of Morf *et al.* was selected for the microarray comparison [29], since the study used the WB isolate and a recent version of the *G. intestinalis* microarray. 6,101 oligonucleotide probes were mapped to the WB genome, of which 5,661 probes mapped unambiguously (mapping quality = 42). 4,619 of the mapped probes were contained in 3,638 protein-coding genes. Raw microarray data were normalized as described by Morf *et al.* [29]. 1,033 genes contained ≥1 probes per gene, only considering probes with normalization *p*<0.05. RNA-seq measurements displayed a moderate but significant correlation with microarray measurements (Figure S4A; *r* = 0.46, *p*<2.2e-16). Comparison with two other microarray data sets from GiardiaDB gave very similar correlation coefficients (data not shown). Gene expression levels were also compared with SAGE data [24], resulting in a weak correlation (Figure S4B; *r* = 0.07, *p* = 9.329e-05). In conclusion, transcription levels measured by RNA-seq had a moderate but significant correlation with microarray measurements but not with SAGE. The latter finding is not surprising since SAGE is generally not quantitative.

**Figure 2:**
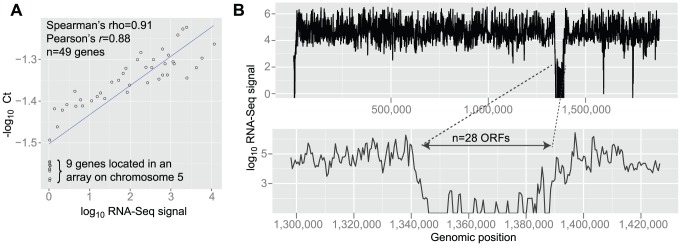
A non-transcribed region on chromosome 5. (A) Correlation of RNA-seq and RT-qPCR gene expression measurements for 49 genes in WB. Black dots are genes. The x- and y-axes show log_10_ FPKM and −log_10_ Ct (cycle threshold). Values were incremented by 1 before log_10_-transformation. Each RT-qPCR reaction was performed in triplicates, and the average Ct was used. The blue line is the linear regression (y = 0.07008x-1.50276). Included genes (prefix GL50803_): 7766, 9662, 6744, 7760, 112103, 11654, 11118, 2661, 24321, 17121, 17585, 14993, 16924, 13272, 6564, 5800, 3367, 17570, 16343, 93548, 11540, 5435, 15000, 21423, 10297, 114210, 86681, 7573, 7715, 102438, 7243, 16438, 17291, 1903, 17495, 102978, 11642, 17539, 90575, 32674, 13091, 137688, 3666, 25075, 16690, 2633, 92664, 13627, 4431. (B) Sliding window analysis of RNA-seq coverage on scaffold CH991767 (part of chromosome 5; WB). Analyzed windows were 500 bp wide and not overlapping. (X-axis) Position along the genomic segment (start position of the analyzed window). (Y-axis) RNA-seq depth in the window on a logarithmic scale. Drop in the RNA-seq coverage is seen in positions 1,340,000 to 1,381,000.

Forty-nine transcripts were evaluated using real-time quantitative PCR (RT-qPCR; Materials and Methods; Gene IDs and primer sequences are listed in Table S2). Genes were selected to represent diverse expression levels (median FPKM = 63): 11 genes (FPKM<0.5), 17 genes (0.5≤FPKM<100), 12 genes (100≤FPKM<1000) and 9 genes (FPKM≥1000). Genes for which FPKM<0.5 were mainly included as a control and most of them were located in a non-transcribed 41 kb region on chromosome 5 (9 genes). The negative logarithm of the RT-qPCR cycle threshold was plotted against the log_10_-scaled FPKM values (Figure 2A). There was a strong correlation between the RNA-seq and RT-qPCR signals (*r* = 0.88, *p*<2.2e-16; Spearman's rho = 0.91, *p*<2.2e-16).

### Culture-induced Biological Variation

It can be hypothesized that gene expression is altered after axenization. We sought to understand what genes displayed altered gene expression in the recently recovered AS175 isolate between *in vitro* passages 4 and 33. We used the fold change of the normalized counts (FPKM) as the metric of gene expression change. The values were incremented by 1 to avoid division by zero. One χ^2^ test was performed for each gene to establish if the observed fold change between *in vitro* passage 4 and 33 was significant. The χ^2^ test (df = 2) called 596 genes significant at α = 0.01 (Table S1; Figure S5). However, it has been shown that less strongly expressed transcripts are more susceptible to Poisson sampling noise [52]; i.e., large fold changes resulting from small fluctuations of RNA-seq coverage. To lower the chance of false positives, we excluded genes expressed at low level, which was here defined as genes with FPKM<50. This resulted in 230 significantly altered genes, of which 95% fell within 1.44 to 2.22X fold change (median = 1.59). 132 genes were more highly expressed after passage 33, e.g. the cell cycle regulatory gene *Wos2* (AS175_2518; 2.67X fold change). Other affected genes were genes encoding transcription factors, metabolic proteins and ribosomal proteins as well as genes specific to *G. intestinalis*. This can reflect the faster growth rate after 33 passages. Next, we categorized differentially expressed genes using Gene Ontology as described in Materials and Methods. Only groups with ≥5 genes were included (GO:0005623, GO:0008150, and GO:0003674 were excluded). None of the ontologies (Biological Process, Molecular Function, and Cellular Compartment) were significant at α = 0.05 (Kruskal-Wallis test; Figure S6). These data indicated that ∼5% of the genes were affected by biological variation and that these genes could not be grouped in a meaningful way. Thus, there are no major gene expression changes between passage 4 and 33 of the AS175 isolate, suggesting that virulence genes expressed *in vivo* also should be expressed *in vitro*. Further studies using parasites isolated directly from the intestine of infected hosts can verify this.

### A Silent Gene Cluster on Chromosome 5

We previously reported shared and isolate-specific genes of WB, P15, and GS [6]. Transcription of the five WB-specific genes could only be weakly detected (FPKM<4). The 38 genes reported to be P15-specific had median expression 0.51 (FPKM). Two of the P15-specific genes exhibited high expression (GLP15_1621 and GLP15_1891; FPKM 1543 and 190). The median of the GS-specific genes was 0.08 FPKM. In conclusion, most but not all isolate-specific genes displayed low expression. While not many genes appear to be stage-specific [24], transcription in a certain life-cycle stage or untested physiological condition cannot be ruled out.

In total, the following numbers of non-transcribed ORFs were identified: 352, 80, 188, and 113 ORFs in WB, AS175, P15, and GS, respectively (FPKM≤0.5). The median lengths of these ORFs were 325, 847, 886, and 804 bp. In WB, 27 non-transcribed ORFs, and one just above the transcription threshold (FPKM = 0.59), were located within a ∼41 kb region on chromosome 5 (CH991767: 1,345,142–1,386,976; Figure 2B). The median length of these ORFs was 958 bp. Two of the ORFs were annotated ‘Spindle pole protein’ (GL50803_92664) and ‘DNA polymerase’ (GL50803_137688). Highly diverged homologs were found in P15 on contig 88, and were without detectable transcription. Extremely low transcription levels were detected using RT-qPCR (Figure 2A), indicating that RNA-seq accurately measured transcription at these ORFs. Sequence signatures of the genes were identified on short contigs in the GS and AS175 genome assemblies. Interestingly, contig 88 is also the site of 13 genes that are shared between P15 and GS, but missing in WB, none of which displayed expression. The complete lack of transcription is surprising, and may suggest a transcriptional blockage, perhaps at the level of chromatin organization. One could hypothesize that low transcription might have been positively selected. The region appears to be present in GS and may therefore have been acquired before the split of the lineages leading to the extant A and B assemblages. High sequence divergence may indicate a lack of purifying selection. Interestingly, Saraiya *et al.* reported one ORF (GL50803_92663) to derive a small non-coding RNA [53].

### Gene Expression Divergence Recapitulates the Known Phylogeny

**Figure 3:**
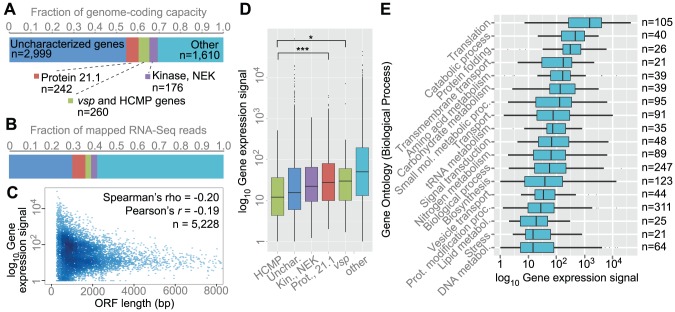
Transcription levels at various classes of protein-coding features. (A) Fraction of the genome occupied by various protein-coding features (WB isolate): uncharacterized genes (hypothetical genes), Protein 21.1, cysteine-rich membrane protein genes (*vsp* and HCMP genes), Kinase NEK and other genes. The x-axis shows the total protein-coding capacity of the genome for each group of genes. (B) Fraction of the RNA-seq data that mapped on categories in (A). (C) Smooth scatter plot of the relationship between gene expression and ORF length. The x- and y-axes show log_10_-scaled FPKM and ORF length (bp). ORFs longer than 8000 bp were not plotted (n = 71). Transition towards more intense blue means higher plotting density. (D) Box plots of gene expression of different categories of genes. Black dots represent outliers. One-way ANOVA concluded a significant difference between the groups (*p*<2.2e-16). The Protein 21.1/HCMP pairwise comparison was significant at *p* = 0.0008972 and *vsp*/HCMP was significant at *p* = 0.04642 (Tukey's HSD test). The groups ‘uncharacterized’ and ‘others’ were ignored in the pairwise statistics. (E) Box plots of genes grouped according to Gene Ontology (GO). Genes were categorized into broader groups by Biological Process using the generic slimmed Gene Ontology. Black dots represent outliers. Numbers to the right indicate how many genes were in the category. Groups are sorted after median expression. The following Gene Ontology categories are shown (GO): 0006412, 0009056, 0006457, 0055085, 0006520, 0005975, 0044281, 0006810, 0006399, 0007165, 0034641, 0008150, 0009058, 0016192, 0006464, 0006629, 0006950, 0006259.

To provide an overview of transcription levels, we counted RNA-seq reads that mapped on various protein-coding features (Figure 3A). Genes were grouped into four categories based on the current annotation: uncharacterized genes (genes without a known function; n = 2,999), Protein 21.1 (n = 242), cysteine-rich membrane proteins (*vsp* and *hcmp* genes; n = 260), Kinase NEK (n = 176) and others (n = 1,610). The large size of the first group reveals how little is known about the biology of this parasite. RNA-seq reads that mapped on ORFs were then counted (Figure 3B). Uncharacterized genes represented 58% of the coding capacity, and accounted for ∼29% of the mapped RNA-seq reads. Protein 21.1, Kinase NEK, and cysteine-rich membrane proteins accounted for 6%, 3%, and 3% of the mapped data. The remaining data mapped onto other genes. There was an approximate inverse relationship between ORF length and gene expression signal (Figure 3C; *r* = −0.19). Longer ORFs tended to exhibit lower transcription levels. As an example, ORFs between 100 to 1000 bp (n = 2,451) had mean expression (log_10_-scaled FPKM) 1.63 and ORFs between 1000 and 2000 bp (n = 1,660) had mean expression 1.45. A qq-plot indicated that the measurements approximately followed a normal distribution, and were significantly different (*t*-test, *p* = 9.047e-10; Wilcoxon signed-rank test, *p* = 4.066e-06). We then compared gene expression signals of genes categorized according to the previous scheme. The analysis indicated wide variation of transcription levels within the groups. None of the groups HCMP, Kinase NEK, Protein 21.1, and *vsp* were significantly different in pairwise comparisons (non-transformed expression values; Tukey's HSD test, *p*>0.05). When gene expression values were logarithmically transformed, the pairwise comparisons of Protein 21.1/HCMP and *vsp*/HCMP became significant (Tukey's HSD test; *p* = 0.0008972, and *p* = 0.04642). We then stratified genes into functional categories using Gene Ontology (Biological Process; Figure 3E). ‘Translation’ had the highest median expression, followed by ‘catabolic process’, and ‘protein folding’. ‘DNA metabolism’ had the lowest median expression. A statistical difference was observed between the groups (one-way ANOVA, *p*<2.2e-16). Each Gene Ontology category displayed a wide range of transcription levels, indicating that genes of these groups are likely not co-regulated.

**Figure 4:**
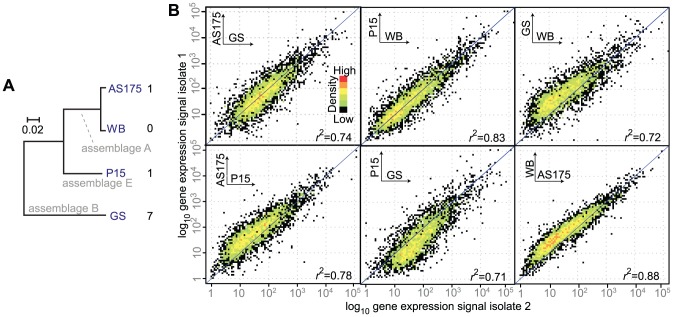
Pairwise comparisons of global transcription levels. (A) Phylogenetic relationship of the four studied isolates. The phylogeny was inferred from 10 concatenated protein-sequences of each genome. The data set was aligned with ClustalW v2.1, and a neighbor-joining tree was constructed using MEGA v.5 [71]. The tree is based on the conceptual translations of the following protein-coding genes (only WB locus-tags are shown; prefix GL50803_): 16936, 31340, 15039, 16445, 14972, 16747, 16681, 9072, 17112, and 11384. The scale bar shows the number of substitutions per site. The name of the isolate is shown in blue at the tip of the branches, and the assemblage (genotype) is shown in light grey. Numbers to the right indicate the number of differentially expressed genes compared to the other isolates. (B) Pairwise correlations of global transcription levels. Each dot represents a conserved four-way ortholog. The x- and y-axes show gene expression of isolate 1 and 2 (log_10_-scaled FPKM; values incremented by 1 before transforming). The colors represent overlap in the plot, i.e. black means a single gene and red means higher plotting density.

The similarity of gene expression between any two isolates was compared. Only orthologs (core genes) were used in the comparisons. Global comparisons of gene expression are shown in Figure 4B and resulted in the following correlation scores (*r* ^2^; each significant at *p*<2.2e-16): 0.74 (AS175 vs. GS), 0.83 (P15 vs. WB), 0.72 (WB vs. GS), 0.78 (AS175 vs. P15), 0.71 (GS vs. P15), and 0.88 (WB vs. AS175). Taken together, these scores recapitulated the known phylogeny of the isolates (a gene based phylogeny is shown in Figure 4A), which indirectly confirmed the accuracy of gene expression measurements. For example, WB and AS175 displayed the most similar gene expression, consistent with these being in the same phylogenetic group (A). Increased phylogenetic distance resulted in increased dissimilarity in gene expression, as seen for example between WB vs. GS and GS vs. P15. The same result was found when genes were stratified into functional groups (Figure S7). Moreover, the asymmetry seen in scatter plots (Figure 4B) indicated directional divergence for the following comparisons: GS vs. WB, AS175 vs. P15, P15 vs. GS, and WB vs. AS175. The pairwise analyses of gene expression indicated a correlation between gene expression and phylogenetic distance. To test this hypothesis across all isolates simultaneously; we computed a distance matrix for gene expression and one another distance matrix for genetic distances. 4175 groups of 1∶1∶1∶1 orthologs were included in the analysis. The two matrices were then tested for a correlation using Mantel's test and 10,000 random permutations, indicating a significant positive correlation between gene expression and genetic distance (*r* = 0.30, *p* = 9.999e-05). These data indicate that gene expression divergence correlates with the phylogeny across the studied isolates.

We sought to determine how many genes were differentially expressed between any two isolates. The fold change of normalized counts (FPKM) was used as the metric of gene expression change. One χ^2^ test (df = 2) for each gene was used to test for significance at α = 0.05. The following numbers of differentially expressed genes were identified: 4 (WB vs. AS175), 12 (WB vs. P15), and 68 (WB vs. GS). See Table S1 for a list of differentially expressed genes. Hence, these data revealed a relatively low number of differentially expressed genes (0.09 to 1.6% of the measured orthologs) and that the counts recapitulated the phylogenetic distance.

### Genome-wide Mapping of Transcript 3′ Ends Using polyA Tags

**Figure 5:**
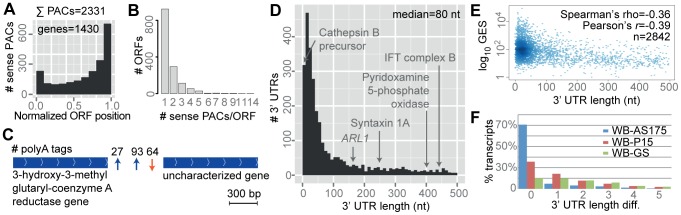
Polyadenylation sites. (A) Histogram of clustered polyadenylation sites (PACs) and their normalized position on ORFs. On the x-axis, 0 and 1 refer to the first and last base of the ORF. Only polyadenylation sites of the sense direction with respect to the ORF are shown. The y-axis shows the frequency. The data are from the WB isolate. (B) Bar-plot of the number of sense PACs per ORF. (C) Example of alternative polyadenylation of the gene encoding 3-hydroxy-3-methylglutaryl-coenzyme A reductase (GL50803_7573). Arrows indicate locations of the polyA site. Blue arrows indicate polyA sites of the same direction as the genes, and the orange arrow indicates a polyA site of the reversed strand. Numbers of supporting reads (polyA tags) are shown on top of the arrows. The polyA sites are separated by 275 bp. (D) Histogram of 3′ UTR length (nt) inferred from mapped polyA sites. Only the WB isolate is shown. (E) Relationship between gene expression signal (GES; log_10_ FPKM) and 3′ UTR length. The y-axis shows the log_10_ GES and x-axis shows the 3′ UTR length (nt). Only 3′ UTRs <500 nt are plotted. (F) Nucleotide length differences between orthologous 3′ untranslated regions.

*G. intestinalis* has very short (1–14 nt) 5′ untranslated regions (UTRs) in mRNAs and ribosomes lack an AUG scanning mechanism [54], [55]. Thus, regulation of gene expression via the 5′UTR is unlikely. However, 3′ UTRs may carry regulatory features like binding sites for small RNAs (e.g., microRNAs) or otherwise affect mRNA stability [56]. Therefore, determination of transcript 3′ ends (polyadenylation sites) is critical for complete understanding of the biology. Previously determined polyadenylation sites (polyA sites) from *G. intestinalis* have suggested short 3′ UTRs [55], but genome-wide analyses and comparisons between isolates have not been performed. In the present study, we performed the following analyses on the ‘polyadenylation landscape’: (*i*) mapping and comparison of a large number of polyadenylation sites (polyA sites); (*ii*) association of polyA sites with the most likely transcript; and (*iii*) comparison of polyA site conservation between isolates. We started the analysis by extracting RNA-seq reads containing polyA tails. Sequence reads containing a consecutive stretch of ≥7 adenines were extracted from the raw data. This resulted in altogether ∼2.5 million reads (polyA tags). Tags were stripped off polyA and adapter sequences, and aligned with the corresponding genome. Only tags with mapping quality >40 were kept. 57% (456,928/802,116) of the polyA tags of WB were mapped with the outlined quality, revealing 49,027 distinct polyA sites (Table 2). Many polyA sites were in very close proximity (<10 nt), representing biological variation, and not truly different sites [57], [58]. PolyA sites located within 10 nt from each other on the same strand were therefore clustered, defining polyA site clusters (PACs). This process resulted in 25,802 clusters of the WB data (median = 1 polyA site/PAC). To increase the signal to noise ratio, we excluded PACs with fewer than 4 polyA tags. This yielded 7,617 PACs containing 95% (432,804/456,928) of the original tags. With respect to the most common polyA site of each PAC, 35% of the PACs (2,648/7,617) were located between genes (intergenic), 31% (2,331/7,617) were in the sense direction to an ORF, and 34% (2,638/7,617) were in the antisense direction to an ORF (only data of WB shown). Figure 5A shows a histogram of relative locations of sense PACs along ORFs. 25% (1,430/5,687) of the ORFs in WB were overlapping 2,331 sense PACs; i.e., 15% (66,588/432,804) of the polyA tags overlapped ORFs in sense. Most ORFs had only one sense PAC per ORF (Figure 5B). It is plausible that sense PACs represent 3′ ends of truncated mRNAs, even if this will give truncated proteins. Another explanation is that sense PACs have resulted from read-through transcription of an upstream ORF. In contrast, antisense PACs might have resulted from run-through transcription of an upstream ORF on the opposite strand or from leaky transcription initiated from cryptic promoters (antisense transcripts).

**Table 2:** Polyadenylation sites.

Isolate	# raw polyA tags	# mapped polyA tags	# polyA tags MAPQ >40^b^	# distinct polyA sites	# polyA clusters (PAC)	# PACs ≥4 tags	# transcripts	Median length (nt)
WB	802,116	724,737	456,928	49,027	25,802	7,617	3,884	80
AS175^a^	398,470	326,038	183,454	37,028	24,566	5,028	3,057	100
P15	816,480	744,785	436,720	51,499	28,286	8,037	3,800	83
GS	155,734	132,142	71,118	22,221	16,473	2,624	1,651	85

a Replicate number 2 (P33).

b PolyA tags mapped with mapping quality >40.

PACs were assigned to the most likely ORF based on mutual proximity. Initially, we assumed a 1∶1 relationship between PAC and ORF, at this stage ignoring the possibility of alternative polyadenylation (one ORF associated with more than one PAC). We did not consider ORFs shorter than 250 bp. For each ORF, the most 5′ PAC was selected, measured from the last nucleotide of the translational stop codon. We selected the most frequently used polyA site of the PAC as the representative polyA site of the transcript. This process associated 3,884 ORFs with PACs (Table 2; Table S1), corresponding to 73% (3,884/5,299) of the protein-coding gene annotations of ≥250 bp length. The median 3′ UTR length of 3,884 ORFs became 80 nt (Figure 5D), which is shorter than the median 3′ UTR length of *Saccharomyces cerevisiae* (104 nt) [59]. 3′ UTRs displayed large size differences, ranging from only a few nucleotides up to several kb. It should be strengthened that while the mapping of polyA sites using the described strategy is precise, the subsequent association with open reading frames may be less precise and it is possible that some of the longer 3′ UTRs may be incorrect.

Median 3′ UTR lengths of the isolates were very similar (Table 2). Genes without an assigned PAC were often transcribed at low levels (median FPKM = 6; median absolute deviation FPKM = 8), suggesting that the number of polyA tags from these genes was below the detection threshold in the present analysis. It can be hypothesized that genes of similar function share regulatory features in their 3′ UTRs and therefore have similar 3′ UTR length. Genes were grouped according to Biological Process, Cellular Compartment, and Molecular Function (only groups with ≥5 genes were considered). There was no difference in 3′ UTR length when genes were grouped according to Biological Process and Cellular Compartment (one-way ANOVA; *p* = 0.2745 and *p* = 0.08487; GO:0008150 and GO:0005623 were excluded). When genes were grouped according to Molecular Function, the null hypothesis could be rejected at *p* = 2.758e-11 (one-way ANOVA; GO:0003674 was excluded). However, post-hoc comparisons did not indicate any significant groups, but GO:0003735 (structural constituents of the ribosome) was at the border (*p* = 0.069, Tukey's HSD test; *p*<0.05 was considered significant). In conclusion, there was no discernible relationship between 3′ UTR length and gene function.

Adjacent genes located on opposite strands (tail-to-tail gene pairs) may give rise to transcriptional overlap. Of the 1,321 tail-to-tail gene pairs of the WB genome, 1,097 had mapped 3′ UTRs. Of these gene pairs, 81% (892/1,097) displayed transcript overlap with the gene on the other strand. Such pervasive transcription is therefore significantly higher in *G. intestinalis* than for example yeast (11.8%) [59]. Next, we compared the 3′ UTR length with the gene expression level. The correlation between the two variables was moderate but significant (Figure 5E; *r* = −0.39, *p*<2.2e-16). This indicated an inverse relationship between gene expression levels and 3′ UTR lengths, i.e. transcripts with longer 3′ UTRs tended to be expressed at lower levels.

3′-rapid amplification of cDNA ends (3′ RACE) was used to validate a selected number of mapped polyA sites. Nine genes were selected from WB and were subjected to 3′ RACE amplification and dye-terminator sequencing. 3′ RACE data were agreeing with predictions from RNA-seq and corroborated the accuracy of the mapped sites (Table 3). PolyA site heterogeneity was observed in the 3′ RACE data, consistent with the above analysis. For example, the genes encoding Ribosomal protein S4 and Median body protein exhibited alternative polyA site usage in both methods. As a second validation, we compared mapped polyA sites with data from the literature. Mok *et al.* reported that *GiUAP* was using two tandem polyA sites, generating two unusually long 3′ UTRs of ∼522 nt and ∼3 kb [60]. PolyA sites corresponding to both the reported 3′ UTRs were identified from our analysis. Que *et al.* reported that the gene *ENC6* had two different polyadenylation sites, resulting in 3′ UTRs of 10 to 20 nt and ∼300 nt [61]. The same study found the former polyA site to be utilized only during encystation [61]. We identified two genes with the annotation *ENC6* in the genome (GL50803_102961 and GL50803_24372), having corresponding polyA sites at ∼2 kb and ∼331 nt from the stop codons. The shorter polyA site of 10 nt was not identified, consistent with the findings of Que *et al.* Moreover, Svärd *et al.* reported a 9-nt 3′ UTR of the *SR-α* (GL50803_14856) gene [62], consistent with our data. Finally, Skarin *et al.* reported the 3′ UTR of *EF-1α* (GL50803_112304) to be 16 nt [63], which was also consistent with our data. In conclusion, RACE experiments and data from the literature supported the authenticity of mapped polyA sites.

**Table 3:** PolyA sites determined using 3′ RACE.

Product description	Locus tag	Gene expression (FPKM)	3′ UTR (RNA-Seq)^a^	3′ UTR (RACE)^b^
Ribosomal protein S4	GL50803_11359	1832	10 (34)	10–34 (10,10,34)
Ornithine decarboxylase	GL50803_94582	220	31	31 (31,31,31)
Gamma giardin	GL50803_17230	5778	28 (6, 15)	28 (28,28,28,28)
Developmentally regulated GTP-binding protein	GL50803_16200	92	117	117 (117,117)
Uncharacterized gene	GL50803_3419	1219	78 (69)	66 (66)
D-tyrosyl-tRNA decarboxylase	GL50803_13832	1097	61 (65)	64–68 (64,64,64,66,68)
Alpha-8 giardin	GL50803_11649	62	263	262 (262)
26S protease regulatory subunit 8	GL50803_17106	1175	11 (7, 14)	7–14 (11,7,14)
Median body protein	GL50803_16343	233	24 (166)	166–168 (166,166,166,168)

a Length in nucleotides. Determined from the most common polyA site. The parenthesis shows alternative polyA sites.

b Length in nucleotides. The parenthesis shows results from individual experimental replicates.

The above analysis resulted in 49% (3,733/7,617; WB) PACs that were not assigned to any transcript using the described approach (orphan PACs). These consisted of altogether 12,511 distinct polyA sites (111,124 polyA tags). 49% (1,852/3,733) of the orphan PACs were in the sense direction with respect to the annotated ORF. Orphan PACs could not have resulted from oligo(dT)-internal priming, since conversion to cDNA was performed using random hexamers. Orphan PACs may indicate 3′ ends of normal, antisense or leaky transcripts. The current data do not allow distinguishing between these types. We hypothesized that orphan PACs of the sense direction represented alternative polyadenylation sites. 599 orphan PACs were intergenic and 1,847 and 1,287 were oriented sense and antisense with respect to the overlapping ORF. Assuming none of the sense and intergenic PACs resulted from antisense transcripts, this gives 1.11 PACs/per ORF (6,330/5,687), suggesting that 1 of 9 transcripts contains an alternative polyadenylation site. One example of such alternative polyadenylation is shown in Figure 5C. Interestingly, the different number of polyA tags reveals that the two mRNA isoforms are not equally expressed. In the particular example the long isoform is more highly expressed, suggesting variable mRNA stability or preferential polyadenylation. In the same fashion, assuming all of the antisense PACs were from antisense transcripts and not 3′ ends of mRNAs, 16% (1,287/7,617) of the polyadenylation sites were estimated derived from antisense transcripts. However, the latter may be an overestimate since some of the antisense PACs may correspond to mRNAs. The many overlapping polyadenylation sites found herein raise concerns about the previous estimate of ∼19% antisense transcription in *G. intestinalis* [30]. This overestimate may be because of the failure to consider overlapping 3′ UTRs.

The positional conservation of polyA sites across isolates was analyzed. When WB and AS175 were compared, 61% (1,690/2,787) of the orthologous polyA sites were of identical distance from the translational stop codon of the corresponding gene. The divergence of polyA sites largely followed the phylogeny of the isolates (Figure 5F). Frequently the 3′ UTR only differed with a few nucleotides. One limitation of the analysis was that we could not exclude the existence of polyA sites in one isolate, since sequencing depth was lower for GS and AS175. Despite this limitation, we found evidence of polyA site-conservation between these isolates.

### Polyadenylation Signals

**Figure 6:**
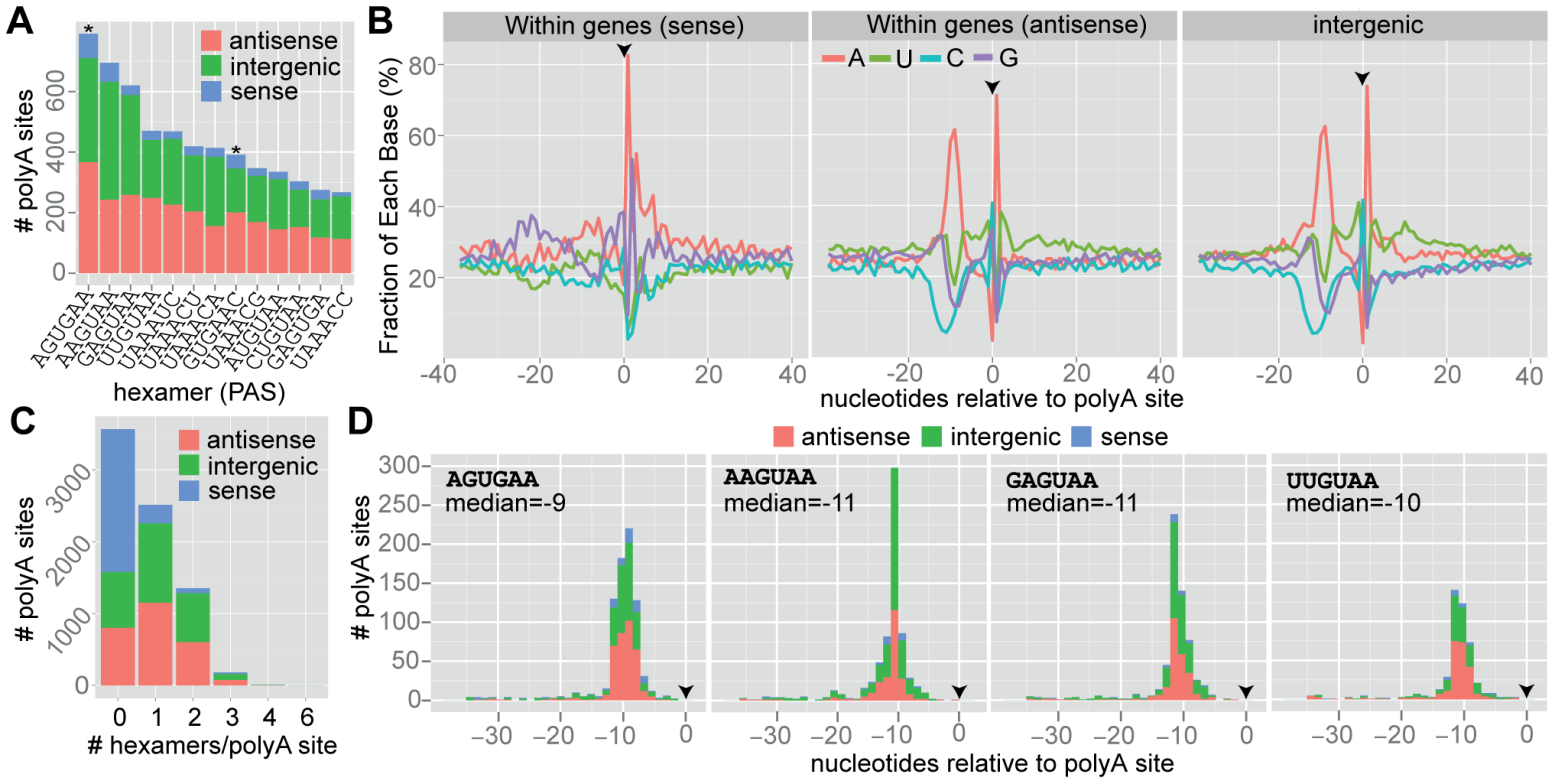
Polyadenylation signals. (A) Prominent hexamer motifs identified in 3′ transcript fragments (data from the WB isolate). The hexamers were identified using the procedure described by Beaudoing *et al.* Positions −40 to −1 relative to the polyadenylation site were searched. One 3′ fragment can contain more than one PAS hexamer and therefore be counted twice. Asterisks (*) indicate hexamers that have previously been reported as putative *G. intestinalis* polyadenylation signals. (B) Nucleotide composition surrounding polyadenylation sites that occur sense, antisense, and intergenic with respect to protein-coding genes. Red, green, blue, and violet correspond to nucleotides A, U, C, and G. The x-axis shows the nucleotide position in relation to the polyA site (black arrow). The y-axis shows the percentage of each base. (C) Frequency of unique hexamers in transcript 3′ fragments (nucleotides −40 to −1 relative to the polyA site). The x-axis shows number of unique motifs found, and the y-axis shows the number of 3′ fragments (split on antisense, sense, and intergenic sites). (D) Histograms of the position of the four most frequent hexamers in relation to the polyA site (black arrow). The position on the x-axis refers to the last nucleotide of the hexamer.

Using the known locations of polyA sites, we systematically searched for putative *cis*-acting elements that may function as polyadenylation signals. In most eukaryotes, the polyadenylation signal is 6 nucleotides long and positioned 10 to 30 nucleotides upstream of the polyA site. In order to identify putative polyadenylation signals, we applied a recursive *k*-mer approach [46] to search the 40 most upstream nucleotides of the polyA site. 7,617 3′ fragments were included in the search, which identified 13 prominent hexamers (WB; Figure 6A). Two of the identified hexamers (motifs) have previously been reported as putative *G. intestinalis* polyadenylation signals (AGUGAA and GUGAAC; [55]), of which AGUGAA was the most common motif identified in our analysis. Several less frequent motifs differed by 1 or 2 nucleotides. None of the motifs were identical with the canonical eukaryotic motif, AAUAAA, although 5 of the 13 motifs contained the tetranucleotide sequence UAAA. The latter tetranucleotide was present in 56.7% (4,319/7,617) of the 3′ fragments. PolyA sites were then grouped into three categories: (*i*) polyA sites located within genes in the sense direction (n = 2,328); (*ii*) polyA sites located within genes in the antisense direction (n = 2,641); and (*iii*) polyA sites located between genes (intergenic; n = 2,648). The positional distribution of the 13 motifs indicated a preferential location at ∼10 nt from the cleavage site (measured from the last nucleotide of the hexamer), which did not differ significantly between motifs and was the same for intergenic and antisense polyA sites (Figure 6D). 53.1% (4,052/7,617) of the 3′ fragments contained at least one of the 13 motifs, and 17.7% (1,351/7,617) of the 3′ fragments contained 2 motifs. Only 2.5% (190/7,617) of the 3′ fragments contained 3 or more motifs (Figure 6C). 46.8% (3,565/7,617) of the polyA sites did not contain any prominent motif. 70.7% (1,873/2,648) of intergenic polyA sites contained one or more motifs and 69.5% (1,837/2,641) of polyA sites located antisense. Only 14.6% (342/2,328) of the polyA sites that were located in the sense direction within a protein-coding gene contained one or more motifs (Figure 6A). Figure 6B shows the nucleotide composition surrounding polyA sites situated sense, antisense, and intergenic with respect to protein-coding genes. Nucleotide composition of intergenic and antisense polyA sites revealed two prominent adenine spikes, located upstream and downstream of the cleavage site. Antisense polyA sites displayed lower content of adenines downstream of the second spike compared with intergenic and sense polyA sites. In contrast, polyA sites that were located sense with respect to protein-coding sequences show only one such adenine spike, present downstream of the cleavage site. The downstream spike of adenines was observed in all three categories and may serve as a prerequisite for binding of the cleavage stimulation factor. The data corroborate previous observations that the polyadenylation signal is embedded in an AU-rich milieu. Noteworthy, 46.8% of the polyA sites of the WB isolate did not contain any prominent polyadenylation signal: sense 85.3% (1,986/2,328); antisense 30.4% (804/2,641); and intergenic 29.2% (775/2,648). The median 3′ polyA tag count of polyA sites with and without any putative polyadenylation signal was 12 and 6, respectively, and the groups were significantly different (Kolmogorov-Smirnov test, *p*<2.2e-16). PolyA sites with one or two motifs had a median tag count of 12 and 14, respectively, and the groups were significantly different (Kolmogorov-Smirnov test, *p* = 0.002702). In conclusion, at least one polyadenylation signal is required for efficient polyadenylation but polyadenylation can occur without any obvious polyadenylation signal.

### Expression of *vsp* Genes

Variant-specific surface proteins (VSPs) are cysteine-rich proteins encoded by *vsp* genes and responsible for antigenic variation in *G. intestinalis*. The haploid genome encodes 200 to 300 *vsp* genes [64], but only one VSP is expressed on the surface of the parasite at any given time [65]. The latter may be due to regulation by RNA interference [21]. We studied the expression spectrum of *vsps* of the WB isolate, since its genome sequence has an accurately assembled *vsp* repertoire. First, we identified *vsps* from the total pool of genomic ORFs using the following criteria: (*i*) the five terminal amino acids had to be one of CRGKA, GRRKA, CRGKL, CRGKL, CHKKA, CRSKA, or YRGKA; (*ii*) the ORF sequence was not allowed to contain any gap or have a gap 300 bp upstream of the start codon; (*iii*) the ORF was located at least 300 bp away from the contig end; and (*iv*) the ORF starts with methionine (ATG). The two latter criteria were employed to avoid including *vsps* for which the 5′ or 3′ ends were incomplete and *vsp* pseudogenes. These criteria identified 185 *vsps*, but 7 pairs of *vsps* were found to have identical protein-coding sequences. Therefore, 178 genes were used for the analysis. Transcription was detected at all of the 178 *vsps* (FPKM ranged from 0.69 to 7271; median = 29 FPKM), agreeing with previous observations. These data suggest that all *vsps* are transcribed in one organism or that *vsp* transcription is heterogeneous in the population.

The consensus sequence PyAatgTT at the beginning of *vsps* (atg refers to the translational start codon) has been suggested to be required for efficient transcription of *vsps* (Inr for initiator sequence; Adam R., unpublished). 70 *vsps* had this consensus sequence at the 5′ end (Inr+) and 108 did not contain the sequence (Inr−). The median and mean FPKM of the Inr+ group was 42 and 169, whereas 19 and 58 for the Inr− group. The two groups were concluded to exhibit different transcription levels (Kolmogorov-Smirnov test, *p* = 7.962e-05). Both groups contained a predominant outlier, which was 3X more strongly expressed in the Inr+ group. The same procedure identified 105 and 69 *vsps* in the genomes of P15 and AS175 respectively, although there was no difference in transcription between the Inr+ and Inr− groups in these isolates (Kolmogorov-Smirnov test; *p* = 0.5449 and *p* = 0.6741). The finding may be due to a smaller sample size, but a biological difference in *vsp* expression cannot be ruled out. We searched for additional DNA motifs in +60 to −20 nt with respect to the first base of the translational start codon, although the search did not reveal any other conserved motif than PyAatgTT. These data indicate that the majority of *vsps* are transcribed and readily detected at the mRNA level, and that the motif PyAatgTT may be important for efficient *vsps* transcription.

### Allele-specific Expression in the GS Isolate

**Figure 7:**
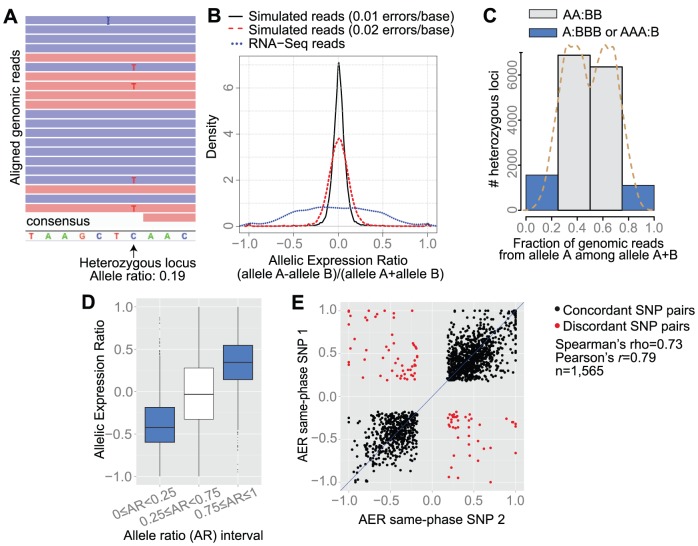
Analyses of allele-specific expression. (A) An example of a heterozygous locus identified from genomic Roche 454 reads (horizontal bars). Colors represent alignments in the forward and reverse directions. The arrow indicates a heterozygous locus. (B) Density plots of allelic expression ratios calculated from simulated reads and RNA-seq reads. The black and red lines correspond to simulated reads containing 0.01 and 0.02 errors/base. The blue line represents RNA-seq data (GS isolate). (C) Histogram of allele ratios (genomic) of heterozygous loci. The superimposed curve (brown) shows the density of the underlying data. The allele ratio was calculated from genomic reads as the fraction of allele A among allele A+B. Grey bars represent loci containing two presumed copies of each allele and blue bars three copies (or vice versa) of each allele. (D) Boxplots of allele expression ratios (y-axis) according to the allele ratio (x-axis). The black line of each box is the median. Dots represent outliers. (E) Allelic Expression Ratios of heterozygous loci of the same haplotype phase. Each dot represents one pair of linked heterozygous loci (SNP pairs). The allelic expression of SNPs 1 and 2 are shown on the x- and y-axes. Red dots indicate discordant heterozygous loci and black dots represent concordant heterozygous loci; i.e., the direction of gene expression change is the same.

*G. intestinalis* trophozoites are tetraploid and therefore contain four alleles of each gene. The GS isolate exhibits ∼0.5% genomic heterozygosity, which is in sharp contrast to the WB and P15 isolates with <0.01% heterozygosity [4]–[6]. Heterozygosity in the GS isolate, and other assemblage B isolates has been shown to occur at the single cell level [66]. The GS isolate therefore provided an opportunity to study allele-specific expression (ASE). We started the analysis by mapping heterozygous loci of GS contigs using the raw genomic reads used to assemble this genome [5]. 19,945 heterozygous positions (loci) were identified in contigs ≥10 kb, of which 80% (15,950/19,945) were located in 1,951 ORFs. Figure 7A shows one heterozygous locus. Since the genome is tetraploid one gene can in theory have four different alleles. One locus contained four different alleles, 36 contained three different alleles and the remaining 15,913 loci contained two different alleles. Only the latter 15,913 loci were used for downstream analyses. Reliable estimation of ASE depends on the accurate alignment of RNA-seq reads with the reference genome. Since the reference sequence contains only one of the alleles, alignments may be biased toward reads containing the reference allele (reference-mapping bias). Various strategies have been proposed to overcome this bias. Vijaya Satya *et al.* used a strategy based on the construction of an enhanced reference sequence, which besides the original reference contains all the alternate alleles [67]. Degner *et al.* reported that masking known SNP positions eliminated the reference mapping bias, but found that 5 to 10% of the SNPs still had an inherent bias toward one allele when using simulated reads of 35 nt length [68]. Degner *et al.* concluded that reference-mapping bias decreased at longer read lengths. Initially, we decided to evaluate the extent of detection bias at the 15,913 heterozygous loci using simulated data. Three copies of the GS genome were created: (*i*) one copy containing allele A; (*ii*) another copy containing allele B; and (*iii*) a third copy with the alleles being masked by a randomly selected, non-allelic nucleotide. Simulated 2×100-nt reads were created from genome copy (*i*) and (*ii*), corresponding to 200X coverage of the masked genome. The simulated reads were generated to follow realistic error profiles (Materials and Methods). The simulation was performed for two sequence error rates: 0.01 errors/base and 0.02 errors/base. Simulated reads from genome copy (*i*) and (*ii*) were then pooled and mapped to the masked genome. An allelic expression ratio (AER) was defined and calculated as the proportion of reads from each allele ((allele A-allele B)/(allele A+allele B); Table S3 lists alleles designated as A and B). AER becomes 0 if the expression is equal, positive if allele A is more highly expressed than B and vice versa. The simulated data showed that there was no systematic bias toward preferential mapping of one allele, as indicated by the symmetrical shape of the curves in Figure 7B. For the simulated data with the error rate 0.01, 97% of the heterozygous loci fell within −0.2≤AER≤0.2. When the error rate was increased to 0.02 errors/base, 91% of the heterozygous loci fell within −0.2≤AER≤0.2. Without bias it would be expected to observe an AER of 0 at all loci; i.e., an equal number of reads from allele A and B. The simulated data therefore indicated that even without allele-specific expression, many heterozygous sites display a bias towards one allele. We decided to estimate the error rate of the RNA-seq data. To do this, we examined aligned RNA-seq reads of the WB isolate. As previously mentioned, WB contains <0.01% genomic heterozygosity, and therefore alignment mismatches are most likely due to sequence errors in the mapped data. 4,257,792 mapped sequences of WB were examined, detecting 1.15 mismatches per aligned sequence read (median = 1), corresponding to an average error rate of 0.01 errors/base. We assumed that the error rate was 0.01 errors/base in the GS RNA-seq data and we used the simulated data with this error rate for significance testing.

Using the real RNA-seq data of GS we explored allelic expression at the 15,913 heterozygous loci. To eliminate mapping bias towards the reference allele, the RNA-seq data of GS were re-aligned with the masked contigs as described in the previous section (contigs ≥10 kb). 13,036 of the 15,913 heterozygotic loci had RNA-seq coverage ≥20X and were used for subsequent analyses (median coverage = 131X). 98% (1,858/1,892) of the examined genes with at least one heterozygous locus had biallelic transcription; i.e., both alleles were detected at the mRNA level. In the same way as for the simulated data, we calculated an allelic expression ratio for the two alleles (A and B). The distribution of allelic expression ratios from RNA-seq and simulated data are shown in Figure 7B, which shows RNA-seq with a more flattened curve compared with simulated data. The flattened appearance may be because of dosage effects caused by different allele counts, or allele-specific expression. A third explanation would be heterogeneous expression in the cell population. The effect was stable at high RNA-seq depths (data not shown), indicating robust statistics. To determine the number of significant loci, we used a normal distribution with mean 0.00944 and standard deviation 0.114 calculated from the simulated data. Two one-sided tests were performed, to test the hypothesis that the AER was less or greater than expected. 8,675 heterozygous loci were significant at α = 0.05, and these sites were distributed over 1,684 genes. Hence, of the 1,892 examined genes with two alleles, 89% (1,684/1,892) of the genes indicated allelic imbalance at the mRNA level. Whether the cause is allele dosage or differences at the transcriptional level, it is clear that many genes have a predominant allele.

We then investigated if there was a correlation between inferred allele count (allele-dosage) and allelic expression. For each heterozygous site, we calculated an allelic ratio defined as: (number of genomic reads from allele A)/(number of genomic reads from allele A+B). At any of the investigated loci, allele A and B can be present in the ratios AA:BB, A:BBB, or AAA:B. The two latter scenarios would in theory result in higher expression of allele B and A, respectively. We divided allele ratios calculated from genomic reads into four equal bins as shown in Figure 7C. The median AER of the bins became: −0.4222 (0≤AR<0.25), −0.2195 (0.25≤AR<0.50), 0.1671 (0.50≤AR<0.75), and 0.3448 (0.75≤AR≤1). Hence, the median AER increased with the allele ratio, and the medians were significantly different (non-parametric Kruskal-Wallis test; *p*<2.2e-16). When the two middle bins were merged (0.25≤AR<0.50 and 0.50≤AR<0.75), their median became −0.0323 (Figure 7D); i.e., equal allele dosage results in equal allele expression. However, it should be emphasized that while the median AER followed the inferred allele count, there was considerable variation of allele expression within each bin. It is unclear if this variation represents methodological noise, biological noises or allele-specific expression. In conclusion, the analyses indicated that 98% of the examined genes had both alleles represented at the mRNA level, and there was a correlation between allele-dosage and expression levels.

The accuracy of the ASE quantifications was investigated using SNP pairs of the same haplotype phase. If ASE was accurately measured, then SNP pairs of the same haplotype and coding sequence should agree on the quantity and direction of ASE, since these are under the same transcriptional control. As previously noted by DeVeale *et al.*, few biological processes are present to disrupt this relationship [69]. We determined SNP pairs of the same haplotype-phase using the raw genomic reads. Only SNP pairs showing ASE *p*<0.05 were used. SNPs were also required to exhibit an allelic ratio of 0.25 to 0.75 in the genome, i.e. assuming that each SNP occurs in two independent alleles. SNPs had to be present inside the same ORF and separated by at least 100 nt. The outlined criteria identified 1,565 pairs of same-phase SNPs that were separated by a median distance of 156 bp. Of these SNP pairs, 6% (101/1,565) completely disagreed on the direction of bias, representing an estimation of the false-positive error rate (discordant SNP pairs; Figure 7E). These data suggest that allelic-expression were accurately measured at the majority of loci, but that a certain level of noise must be accounted for, which is also consistent with previous studies [69].

Our interpretation of the current data is that it confirms biallelic expression at almost all examined loci, and that it shows a correlation between allele expression and allele dosage. These data suggest that binucleic transcription is symmetric in general. The large number of protein isoforms that are potentially present at the protein level may have an impact on biological variation and fitness.

### Genome-wide Splice Junction Mapping Identifies Only One Novel Intron

Relatively few introns have been documented in *G. intestinalis*: (*i*) Nixon *et al.* reported a 35-bp spliceosomal intron in the gene encoding [2Fe-2S] ferredoxin [20]; (*ii*) Russell *et al.* reported an intron in *Rpl7a* and another one in an uncharacterized gene (GL50803_35332) [19]; (*iii*) Morrison *et al.* reported an intron in the gene encoding a dynein light-chain protein (GL50803_15124) [4]; and (*iv*) Roy *et al.* found an intron in an Rpn10 homolog (GL50803_15604) [17]. In the present study, we searched for additional splice junctions using the software TopHat [49]. We then performed cross-isolate comparisons of the mapped junctions to find new candidates. TopHat listed 510, 402, 598, and 214 putative splice junction pairs of WB, AS175, P15, and GS, respectively. Three out of the five reported introns were identified in all 4 isolates, and two of the reported introns (in the genes GL50803_35332 and GL50803_15124 respectively) were identified in 2 and 3 isolates, respectively. The intron in GL50803_35332 was detected in P15 and AS175, and had only one (AS175) and three (P15) supporting reads. The gene was fragmented in the GS genome assembly and presumably below the level of detection in WB. With this in mind, we assembled a list of all putative splicing events that adhered to the following criteria: (*i*) splicing is supported by at least two isolates; (*ii*) the implicated gene is not repeated; i.e., part of a gene family (e.g., *vsp*, HCMP, and Protein 21.1); and (*iii*) splicing is supported by at least 5 reads. These criteria identified 14 intron candidates that were further investigated by PCR on genomic DNA and RNA (cDNA) from WB trophozoites. The PCR validation rejected 13 of the 14 candidates (Figure S8), suggesting that splice junction mapping with RNA-seq is noisy. The validated intron was 36 nucleotides long and situated upstream of the GL50803_86945 gene (an uncharacterized gene), and it was conserved in all four isolates. Removal of the intron extended the open reading frame of GL50803_86945 with 73 codons. The size of the predicted intron (36 nt) was further consistent with the result from agarose gel electrophoresis, and the splice site was confirmed by dye-terminator sequencing of the cDNA PCR product. Comparison of the 5′ to 3′ splice boundary with the known introns revealed a canonical splice site (GT-AG) (Figure S8). Five out of the six introns displayed positional bias towards the 5′ end of the gene.

The present study confirms the previously reported *G. intestinalis* introns and identifies one novel intron. Finally, it is possible that some genes transcribed at very low levels contain unidentified introns since the criteria used here were conservative. However, the true number of introns in *G. intestinalis* is unlikely to be much higher than reported here.

### Concluding Remarks

In the present study we describe global analyses of the polyadenylated transcriptome of four *G. intestinalis* isolates, representing three distinct assemblages (syn. genotypes; A, B, and E). Gene expression was measured in trophozoites grown *in vitro*, which revealed transcription at ∼99% of the conserved open reading frames (ORFs). This confirms the promiscuous nature of transcription in this parasite, revealing that almost the entire genome is transcribed in trophozoites. In addition to providing transcriptional evidence for the majority of gene models, the data were also used to perform an evolutionary comparison of gene expression. This found that expression divergence recapitulated the known phylogeny of these strains. This observation suggests that gene expression in this species has largely evolved via neutral drift followed by purifying selection. These data are much in line with findings in other organisms. However, the question of how *G. intestinalis* maintains different gene expression levels is still unresolved. Because of the extensive sequence divergence between the A, B, and E assemblages it is unlikely that gene expression is to any greater extent regulated at the transcriptional level. The current study provides some examples of genes with lineage-specific expression, which may highlight traits or features that are prominent in one isolate compared to others. However, the functional importance of these differences remains to be investigated.

We mapped a large number of polyadenylation sites, providing the first global picture of the ‘polyadenylation landscape’ of this parasite. These data revealed many instances of unexpectedly long 3′ untranslated regions (UTRs), and an abundance of orphan polyadenylation sites, which do not seem to belong to mRNA. The latter suggests pervasive transcription, alternative polyadenylation, or transcriptional noise. Interestingly, 3′ UTR lengths have largely been conserved across the three assemblages, suggesting that it may serve roles in transcript regulation or convey regulatory signals. Using mapped polyadenylation sites we searched for putative polyadenylation signals. Several novel motifs were identified and overrepresented at the 3′-end of the transcript, although none of these signals were identical with the canonical eukaryotic polyadenylation signal. However, the tetranucleotide sequence ‘UAAA’ was shared by several motifs, and this tetranucleotide sequence is also a part of the canonical eukaryotic polyadenylation signal (AAUAAA). These data suggest that the *G. intestinalis* polyadenylation signals may be more similar to the canonical eukaryotic ‘AAUAAA’ than previously recognized. Moreover, a subset of genes was missing any clear motif, suggesting that a strict polyadenylation signal may not be required for proper 3′-end formation. This finding is consistent with higher eukaryotes.

The previously demonstrated heterozygosity of GS [5] was found manifested at the transcriptional level along with evidence of allelic imbalance that was correlated to allele dosage, contributing to an expanded repertoire of protein isoforms of this *G. intestinalis* isolate. Since the present study is performed on a population of parasites, it is possible that allelic imbalance is due to heterogeneous expression in the population. Moreover, we performed a global mapping of splice junctions and found all the previously reported introns as well as one new. These data suggest that *G. intestinalis* is unlikely to have many more introns than presented here. The presence of the six introns is enigmatic and it is tempting to speculate that the parasite has either undergone intron loss or that introns have been sparse in this eukaryote. Notably, the novel intron was located 5′ to the annotated gene, similar to four of the previously documented introns. The same bias is seen in intron deficient protozoa that belong to intron-rich groups of organisms [70], suggesting that intron loss is the most likely explanation for *G. intestinalis*.

## Supporting Information

Figure S1 **Fragment size distributions inferred from mapped data.** Fragment size histograms for each sequencing library. The x-axis shows the fragment size in nucleotides, and the y-axis shows the number of read-pairs. Fragment sizes were determined from mapped data. The fragment size was defined as the distance in nucleotides between the left-most position of the left read to the right-most position of the right read.
(PDF)

Figure S2 **Estimates of technical variation.** Scatter plots of technical replicates from each sequencing library. Each dot represents a gene. The x-axis shows log_10_-scaled FPKM values from lane 1, and the y-axis shows log_10_-scaled FPKM values from lane 2. FPKM values were incremented by 1 to avoid infinite values. The Pearson's *r* ^2^ is shown in the top left corner and indicated very low technical variation.
(PNG)

Figure S3 **Average fold coverage of RNA-seq data on ORFs.** Average fold coverage of reads on ORFs. Only reads from the same strand were included for coverage computation (i.e., antisense transcription was excluded). Only four way orthologs were included. The y-axis shows the average fold coverage, and the x-axis shows the distance in nucleotides from the 5′ and 3′ ends respectively. Average fold coverage was determined as follows: For each ORF the average coverage on the same strand was computed (total coverage on the ORF/ORF length). Subsequently, the fold coverage was calculated for each nucleotide position (coverage of the position/average coverage of the ORF). Finally, the average of the fold coverage was calculated for each position over all ORFs. Only the first 7000 positions from 5′ and 3′ ends were plotted.
(PDF)

Figure S4 **Comparison of RNA-seq with microarray and SAGE measurements.** Comparison of gene expression values computed using RNA-seq with gene expression measurements from microarray (A) and SAGE (B). Correlation coefficients are in the bottom right corners as well as number of included genes.
(PDF)

Figure S5 **Differentially expressed genes of the AS175 isolate.** Scatter plot of differentially of gene expression (y-axis = AS175 biological replicate 1, x-axis = AS175 biological replicate 2). Each dot represents a gene. Dots closer to the black line means more similar expression in the two samples. Pink dots are genes that displayed significant fold change compared with the technical replicates (*p*<0.01) but were lowly transcribed. Blue dots are significant genes (*p*<0.01) that were highly transcribed. The latter genes were used for Gene Ontology analysis. Grey dots represent non-significant genes.
(PDF)

Figure S6 **Gene Ontology analysis of differentially expressed genes.** Gene Ontology categorization of genes that were differentially expressed of the AS175 isolate (replicate 1 and 2). Genes were grouped using GO annotations of the four ontologies: Molecular Function, Cellular Compartment, and Biological Process. Gene expression fold change is displayed on the x-axis. The number of genes in each category is shown on the right margin. None of the groups were significant (one-way ANOVA; *p*>0.05). (A) Molecular Function (GO). (B) Cellular Compartment (GO). (C) Biological Process (GO).
(PDF)

Figure S7 **Gene expression divergence for various categories of genes.** Heat-map of gene expression divergence of groups of orthologous genes. The gene expression of each group was compared with Pearson's *r* (0 to 1). Color transitions toward red indicate lower correlation and colors toward white indicate higher correlation. Only categories with ≥5 genes are shown. (A) Genes grouped according to Biological Process. The x- and y-axes show what two isolates are compared and the feature group. The following GO categories are shown (GO:00): 44281, 06810, 06412, 07165, 09058, 06399, 05975, 08150, 06464, 06259, 51276, 06520, 34641, 06461, 34655, 07010, 06950, 06397, 51186, 42254, 16192, 06790, 42592, 06091, 55085, 06457, 09056, 06629, and 06913 (B) Genes grouped according to Molecular Function. The following GO categories are shown (GO:00): 43167, 03674, 03677, 16887, 08135, 03924, 16301, 16791, 16757, 30234, 16491, 16779, 03735, 08168, 51082, 08289, 16829, 22857, 16874, 04386, 08233, 08565, 05198, 16853, 04518, 03723, 04871, 01071, and 16746 (C) Gene families (Protein 21.1; uncharacterized genes; Kinase NEK). (D) Genes with signal peptide and *trans*-membrane domains.
(PDF)

Figure S8 **PCR validation and analysis of** ***cis*** **-intron candidates.** (A) Putative *cis*-introns in the genes GL50803_93294, GL50803_113677, and GL50803_32999 were investigated by PCR of genomic DNA (gDNA) or complementary DNA (cDNA) from *G. intestinalis* WB trophozoites. PCR products were separated on a 1% 1xTAE agarose gel and stained with ethidium bromide. GeneRuler 1 kb (Fermentas) and GeneRuler 100 bp ladders (Fermentas) were loaded to allow sizing of PCR products. (B) Putative *cis*-introns in the genes GL50803_6171, GL50803_9861, GL50803_86945, GL50803_17227, and GL50803_5517 were investigated as in A, but PCR products were separated on a 2% 1xTAE agarose gel. GeneRuler 100 bp ladder was loaded to allow sizing of fragments. (C) Putative *cis*-introns in the genes GL50803_16431, GL50803_13864, GL50803_14019, GL50803_103855, GL50803_5359, GL50803_10311 were investigated as in B. PCR amplifications using cDNA reaction with reverse transcriptase omitted (-RT) were performed for the GL50803_86945 and GL50803_13864 primer pairs. (D) Alignments of validated *cis*-introns. The novel intron has extensive similarities at 5′ and 3′ intron splice-borders to known *G. intestinalis* introns. (E) MEME was used to create logos for the 5′ intron and 3′ intron border sequences. (F) List of used primer sequences (5′ to 3′) (G) Alignment of the 5′ end of the gene. The intron is underlined.
(PDF)

Table S1 **Gene coordinates, transcription levels, and differentially expressed genes.** Gene annotation coordinates, digital gene expression values (FPKM), mapped 3′ UTRs, and differentially expressed genes. The first sheet shows details for genes defined as four-way orthologs. Genes that could not be assigned as four-way orthologs are on separate sheets according to isolate. Genes identified as differentially expressed in the biological replicates of AS175 are shown on a separate sheet. Differentially expressed genes are listed for the comparisons: WB vs. AS175, WB vs. P15, and WB vs. GS. The last sheet contains information about identified lineage-specific gene expression.
(XLSX)

Table S2 **RT-qPCR primer list.** A primer list for real-time quantitative PCR used for validation of gene expression calculations.
(XLSX)

Table S3 **Allelic coordinates and expression ratios.** Coordinates and expression data of heterozygous loci of the GS genome.
(XLSX)
