# Supplementary figures and images for: Transcriptome Profiling of Giardia intestinalis Using Strand-specific RNA-Seq
Source: PLoS Comput Biol. 2013 Mar 28;9(3):e1003000. doi: 10.1371/journal.pcbi.1003000 (PMC3610916; doi:10.1371/journal.pcbi.1003000)

Schematic alignment examples:

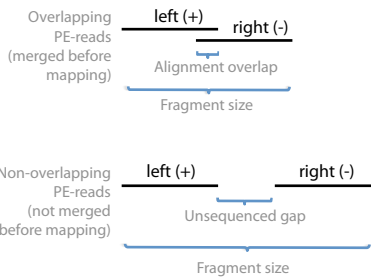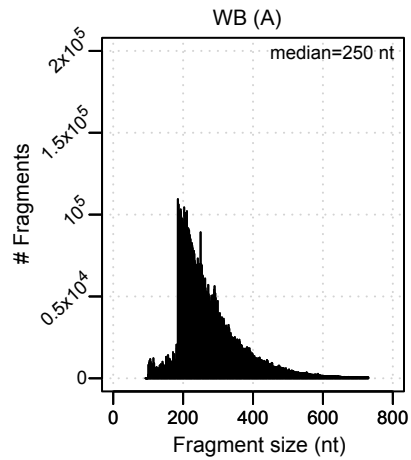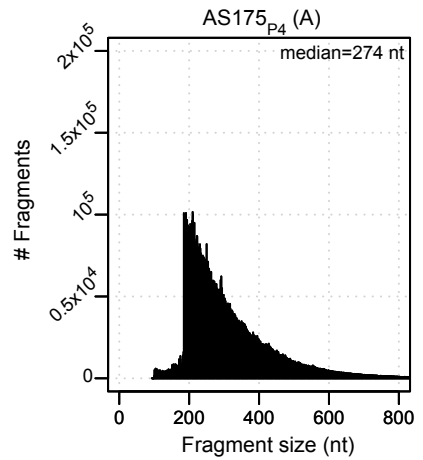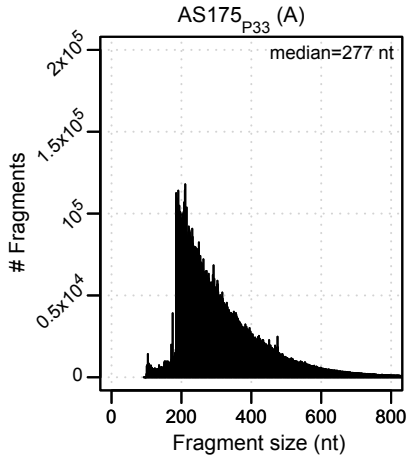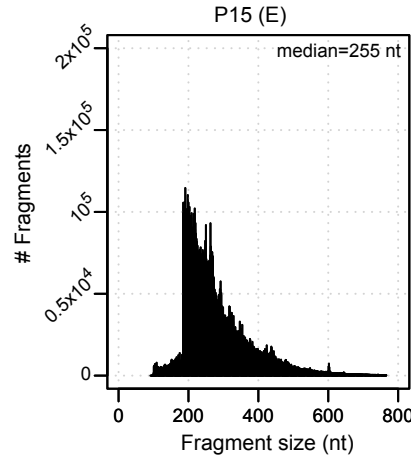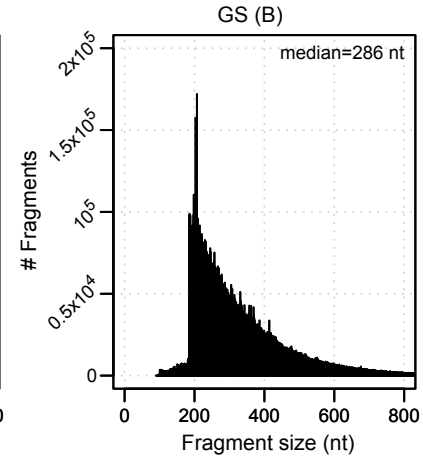

Supplement: Figure S1 — Fragment size distributions inferred from mapped data. Fragment size histograms for each sequencing library. The x-axis shows the fragment size in nucleotides, and the y-axis shows the number of read-pairs. Fragment sizes were determined from mapped data. The fragment size was defined as the distance in nucleotides between the left-most position of the left read to the right-most position of the right read. (PDF) [file pcbi.1003000.s001.pdf]

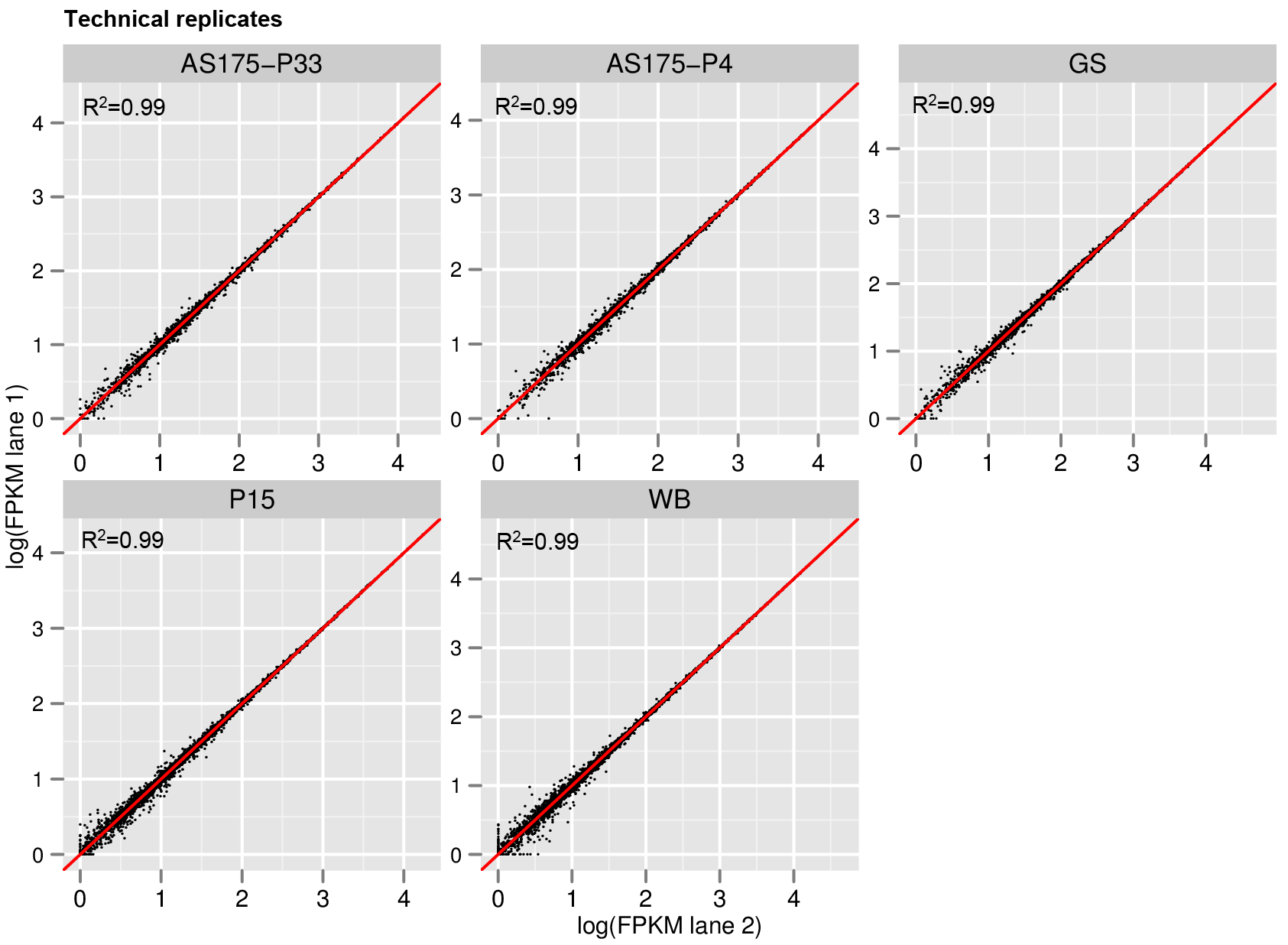

Supplement: Figure S2 — Estimates of technical variation. Scatter plots of technical replicates from each sequencing library. Each dot represents a gene. The x-axis shows log10-scaled FPKM values from lane 1, and the y-axis shows log10-scaled FPKM values from lane 2. FPKM values were incremented by 1 to avoid infinite values. The Pearson's r 2 is shown in the top left corner and indicated very low technical variation. (PNG) [file pcbi.1003000.s002.png]

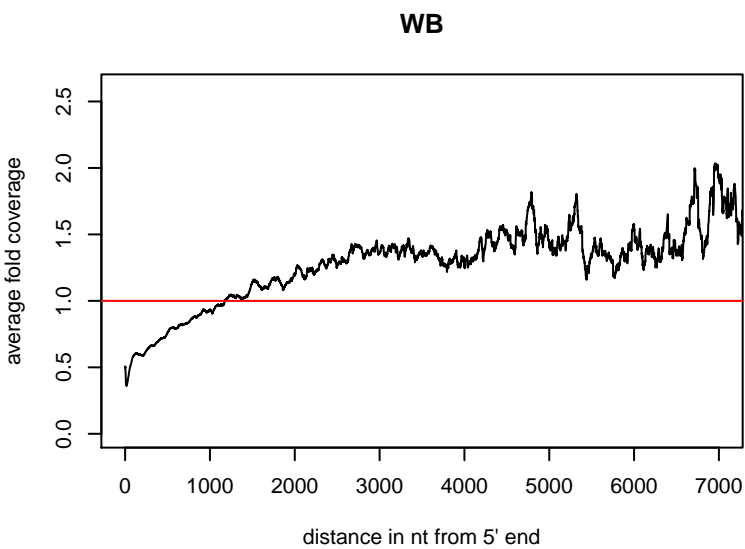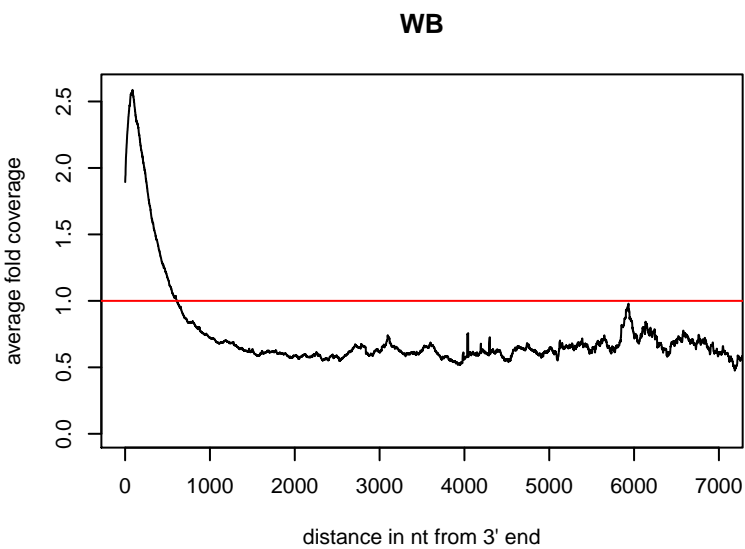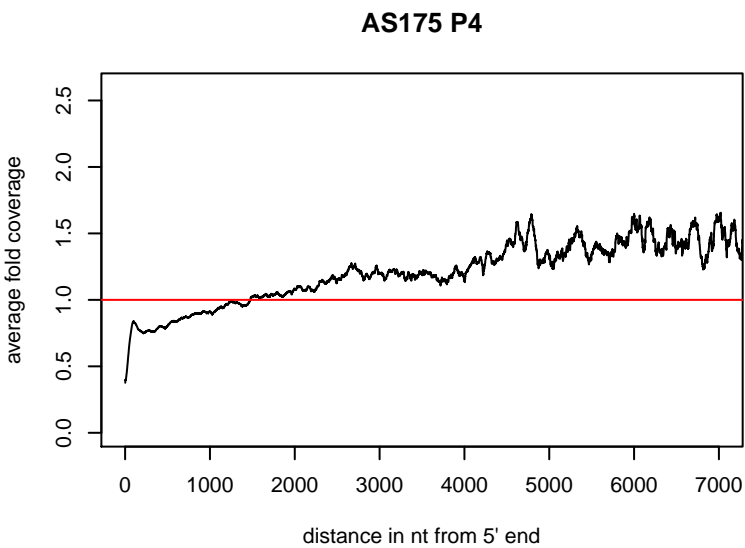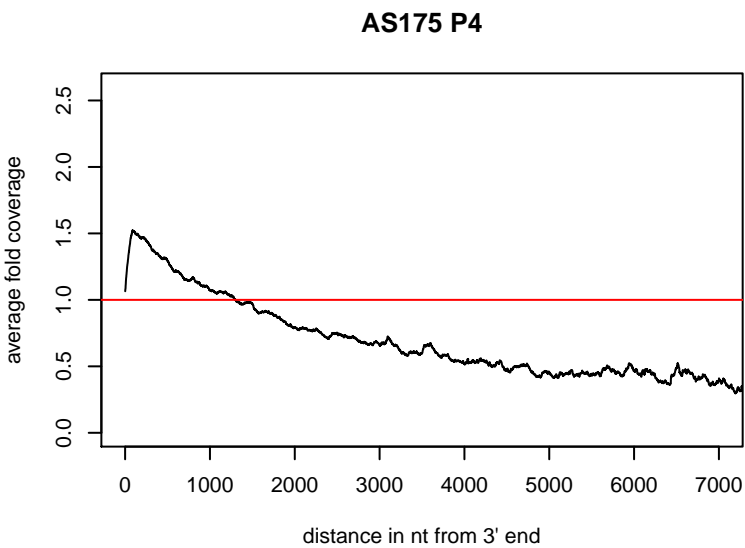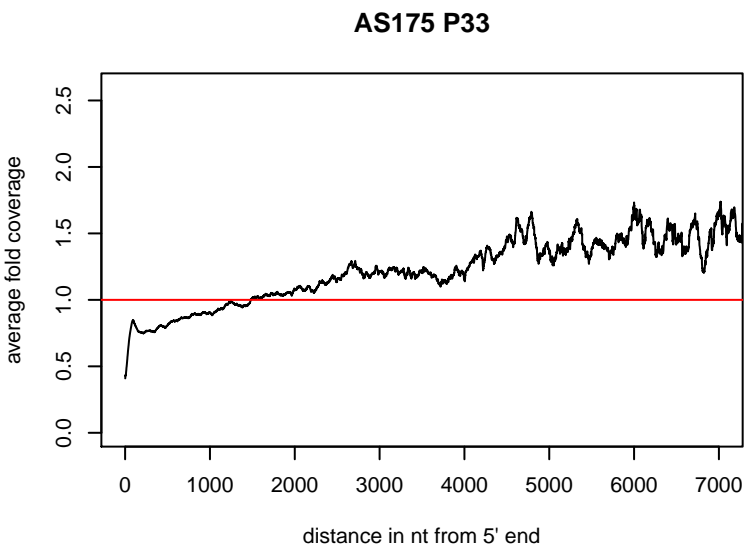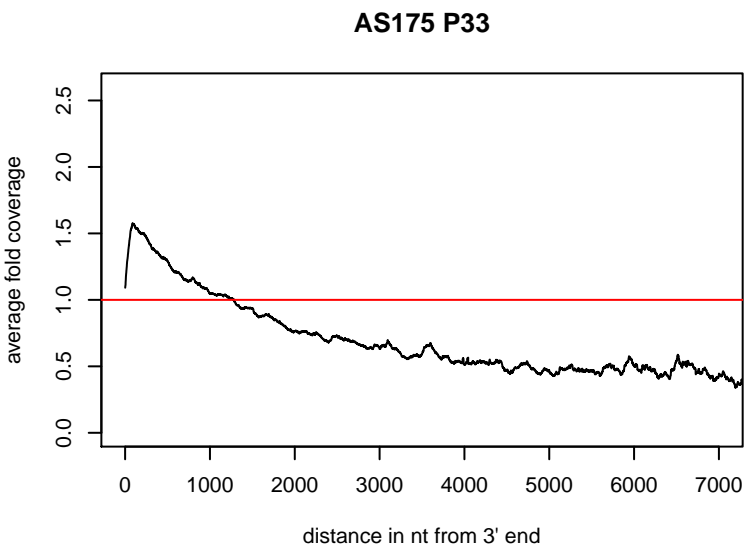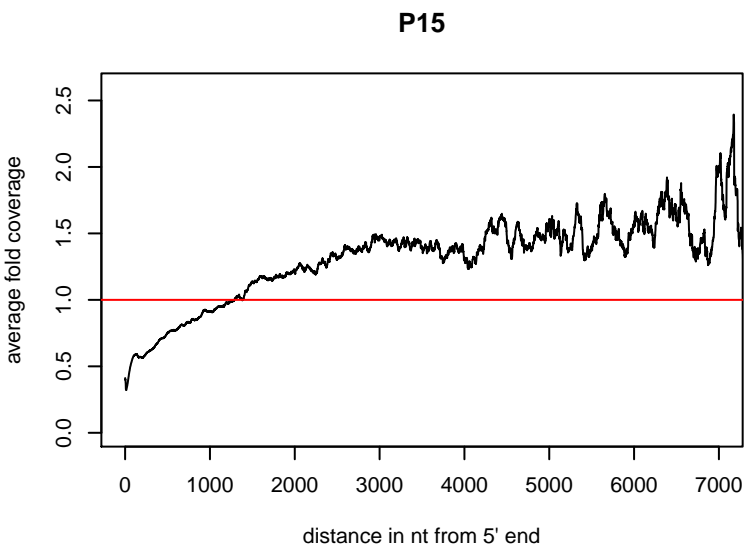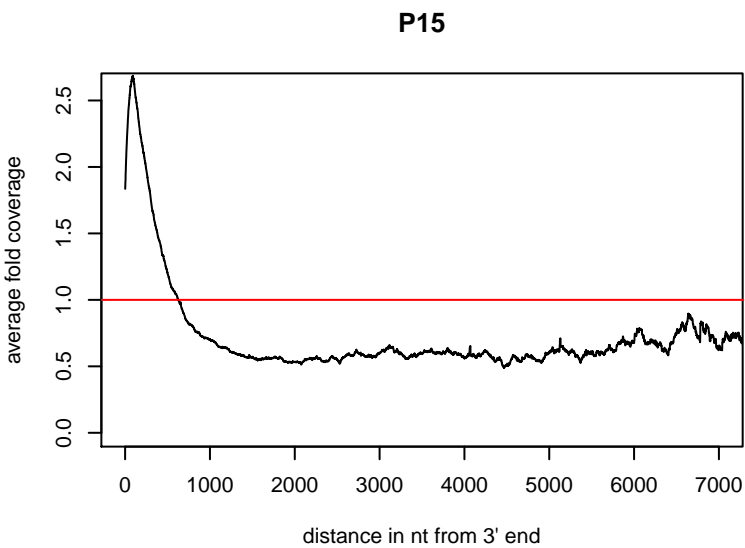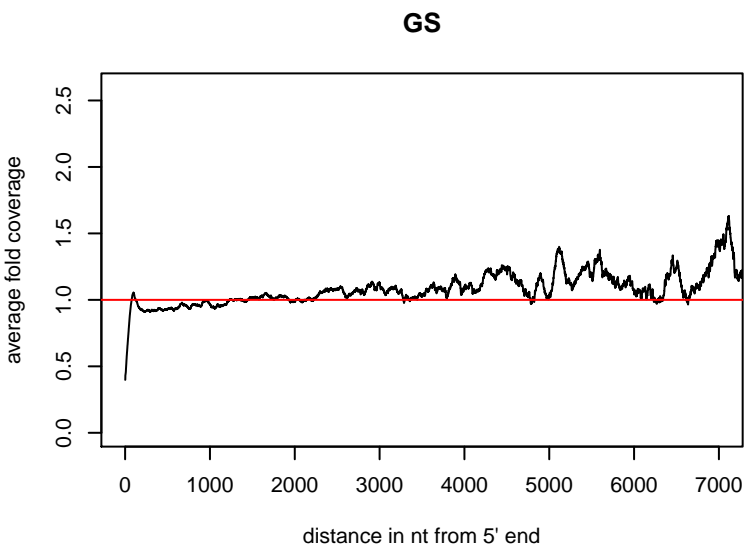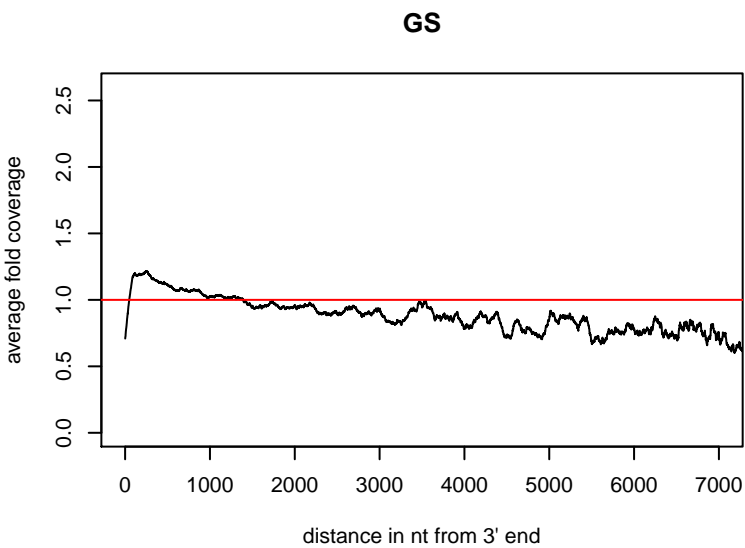

Supplement: Figure S3 — Average fold coverage of RNA-seq data on ORFs. Average fold coverage of reads on ORFs. Only reads from the same strand were included for coverage computation (i.e., antisense transcription was excluded). Only four way orthologs were included. The y-axis shows the average fold coverage, and the x-axis shows the distance in nucleotides from the 5′ and 3′ ends respectively. Average fold coverage was determined as follows: For each ORF the average coverage on the same strand was computed (total coverage on the ORF/ORF length). Subsequently, the fold coverage was calculated for each nucleotide position (coverage of the position/average coverage of the ORF). Finally, the average of the fold coverage was calculated for each position over all ORFs. Only the first 7000 positions from 5′ and 3′ ends were plotted. (PDF) [file pcbi.1003000.s003.pdf]

**A** Microarray vs RNA-Seq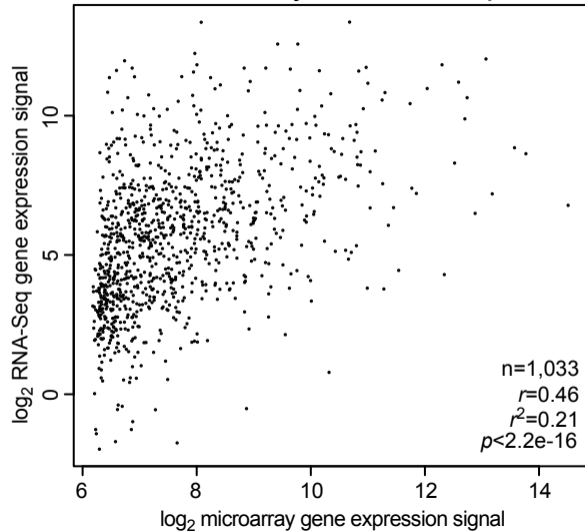**B** SAGE vs RNA-Seq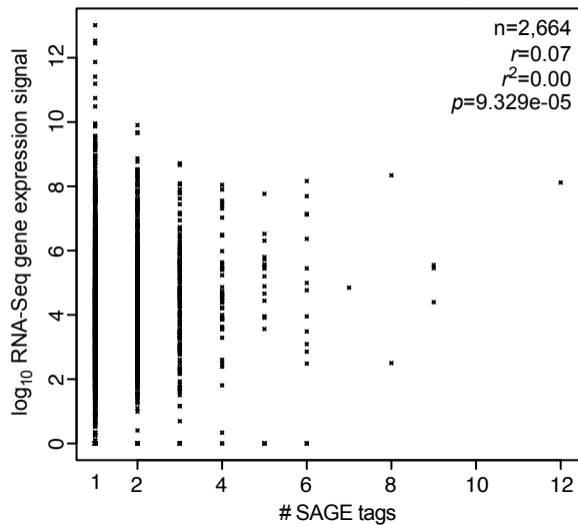

Supplement: Figure S4 — Comparison of RNA-seq with microarray and SAGE measurements. Comparison of gene expression values computed using RNA-seq with gene expression measurements from microarray (A) and SAGE (B). Correlation coefficients are in the bottom right corners as well as number of included genes. (PDF) [file pcbi.1003000.s004.pdf]

Strain AS175 Biological replicates correlation

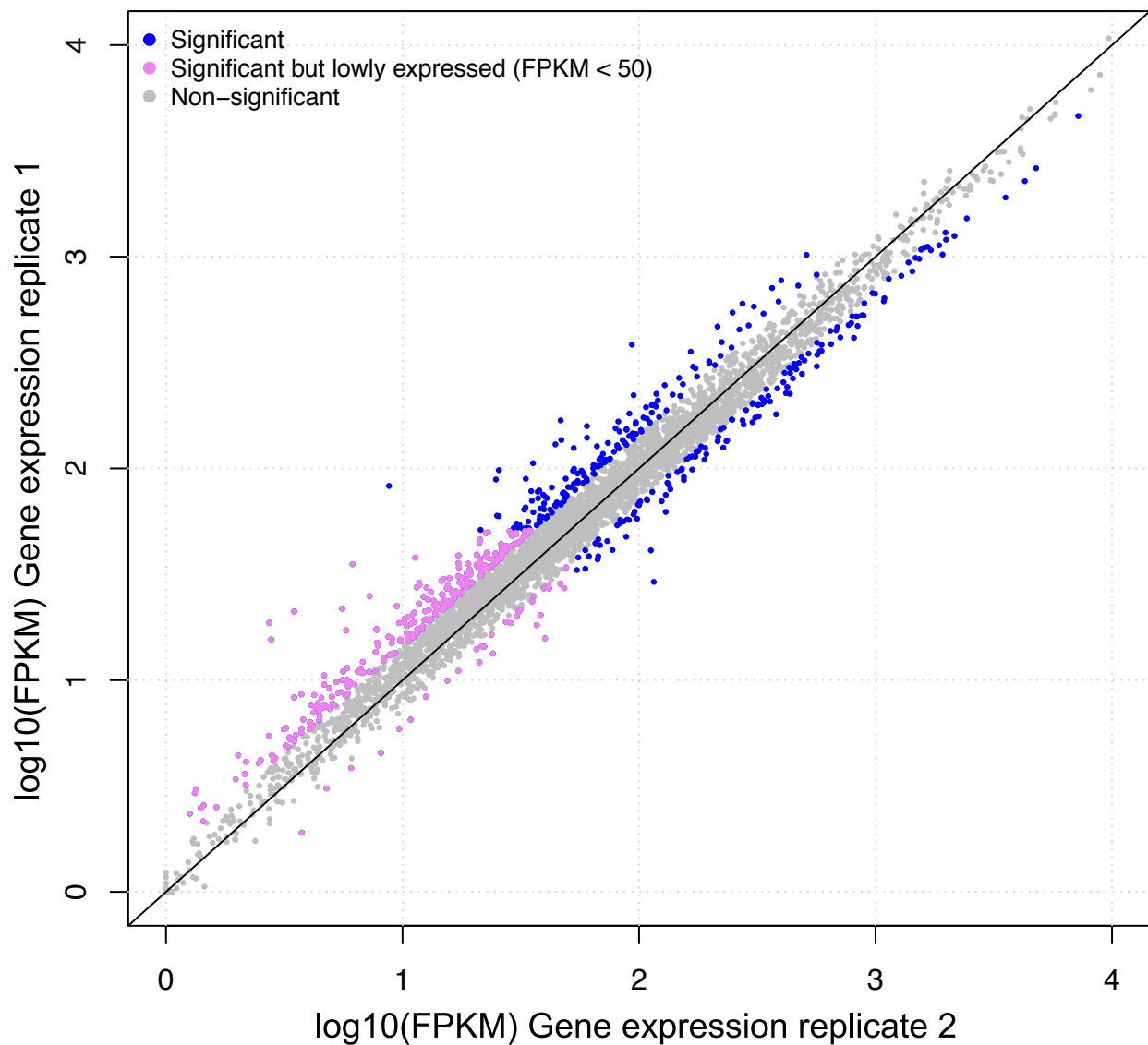

Supplement: Figure S5 — Differentially expressed genes of the AS175 isolate. Scatter plot of differentially of gene expression (y-axis = AS175 biological replicate 1, x-axis = AS175 biological replicate 2). Each dot represents a gene. Dots closer to the black line means more similar expression in the two samples. Pink dots are genes that displayed significant fold change compared with the technical replicates (p<0.01) but were lowly transcribed. Blue dots are significant genes (p<0.01) that were highly transcribed. The latter genes were used for Gene Ontology analysis. Grey dots represent non-significant genes. (PDF) [file pcbi.1003000.s005.pdf]

### A Cellular Compartment

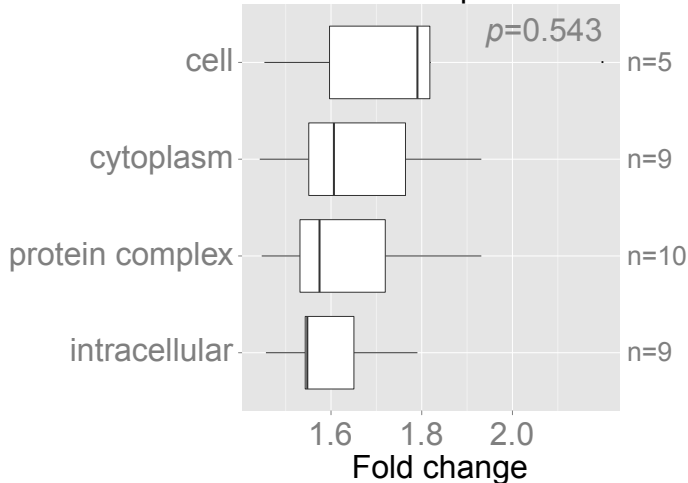

### B Molecular Function

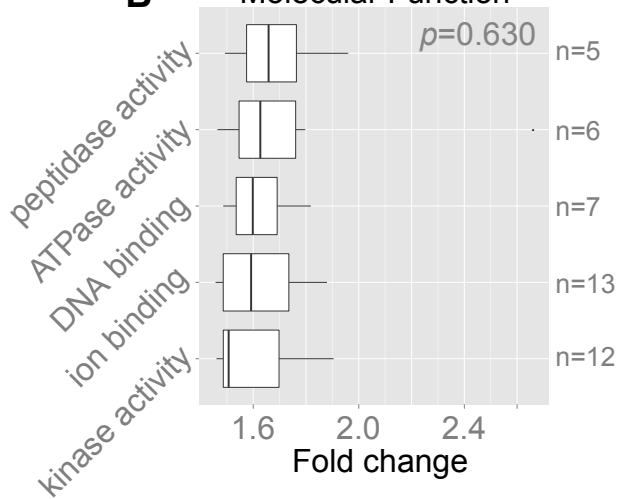

### C Biological process

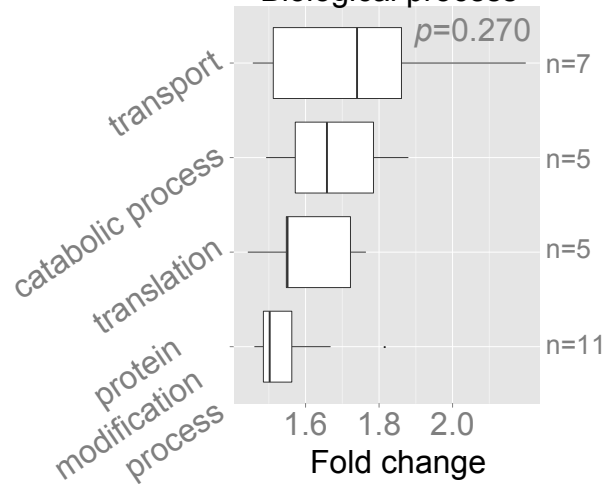

Supplement: Figure S6 — Gene Ontology analysis of differentially expressed genes. Gene Ontology categorization of genes that were differentially expressed of the AS175 isolate (replicate 1 and 2). Genes were grouped using GO annotations of the four ontologies: Molecular Function, Cellular Compartment, and Biological Process. Gene expression fold change is displayed on the x-axis. The number of genes in each category is shown on the right margin. None of the groups were significant (one-way ANOVA; p>0.05). (A) Molecular Function (GO). (B) Cellular Compartment (GO). (C) Biological Process (GO). (PDF) [file pcbi.1003000.s006.pdf]

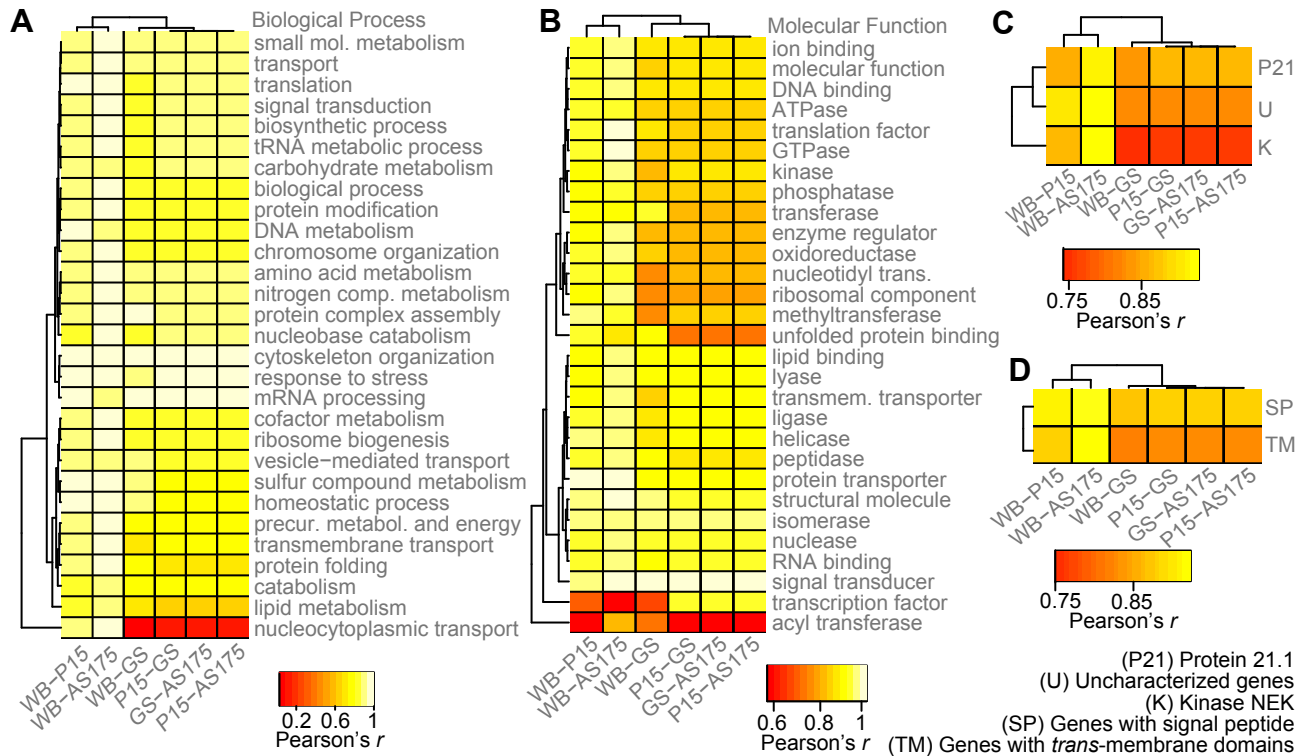

Supplement: Figure S7 — Gene expression divergence for various categories of genes. Heat-map of gene expression divergence of groups of orthologous genes. The gene expression of each group was compared with Pearson's r (0 to 1). Color transitions toward red indicate lower correlation and colors toward white indicate higher correlation. Only categories with ≥5 genes are shown. (A) Genes grouped according to Biological Process. The x- and y-axes show what two isolates are compared and the feature group. The following GO categories are shown (GO:00): 44281, 06810, 06412, 07165, 09058, 06399, 05975, 08150, 06464, 06259, 51276, 06520, 34641, 06461, 34655, 07010, 06950, 06397, 51186, 42254, 16192, 06790, 42592, 06091, 55085, 06457, 09056, 06629, and 06913 (B) Genes grouped according to Molecular Function. The following GO categories are shown (GO:00): 43167, 03674, 03677, 16887, 08135, 03924, 16301, 16791, 16757, 30234, 16491, 16779, 03735, 08168, 51082, 08289, 16829, 22857, 16874, 04386, 08233, 08565, 05198, 16853, 04518, 03723, 04871, 01071, and 16746 (C) Gene families (Protein 21.1; uncharacterized genes; Kinase NEK). (D) Genes with signal peptide and trans-membrane domains. (PDF) [file pcbi.1003000.s007.pdf]
