# Supplementary material for: Transcriptome Profiling of Giardia intestinalis Using Strand-specific RNA-Seq
Source: PLoS Comput Biol. 2013 Mar 28;9(3):e1003000. doi: 10.1371/journal.pcbi.1003000 (PMC3610916; doi:10.1371/journal.pcbi.1003000)
Supplement: Figure S8 — PCR validation and analysis of cis -intron candidates. (A) Putative cis-introns in the genes GL50803_93294, GL50803_113677, and GL50803_32999 were investigated by PCR of genomic DNA (gDNA) or complementary DNA (cDNA) from G. intestinalis WB trophozoites. PCR products were separated on a 1% 1xTAE agarose gel and stained with ethidium bromide. GeneRuler 1 kb (Fermentas) and GeneRuler 100 bp ladders (Fermentas) were loaded to allow sizing of PCR products. (B) Putative cis-introns in the genes GL50803_6171, GL50803_9861, GL50803_86945, GL50803_17227, and GL50803_5517 were investigated as in A, but PCR products were separated on a 2% 1xTAE agarose gel. GeneRuler 100 bp ladder was loaded to allow sizing of fragments. (C) Putative cis-introns in the genes GL50803_16431, GL50803_13864, GL50803_14019, GL50803_103855, GL50803_5359, GL50803_10311 were investigated as in B. PCR amplifications using cDNA reaction with reverse transcriptase omitted (-RT) were performed for the GL50803_86945 and GL50803_13864 primer pairs. (D) Alignments of validated cis-introns. The novel intron has extensive similarities at 5′ and 3′ intron splice-borders to known G. intestinalis introns. (E) MEME was used to create logos for the 5′ intron and 3′ intron border sequences. (F) List of used primer sequences (5′ to 3′) (G) Alignment of the 5′ end of the gene. The intron is underlined. (PDF) [file pcbi.1003000.s008.pdf]

A.

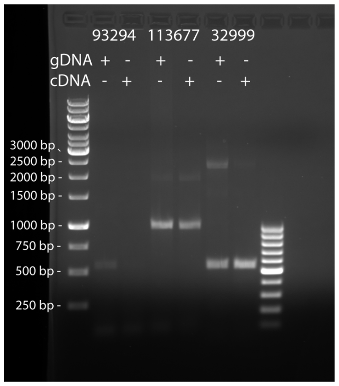

B.

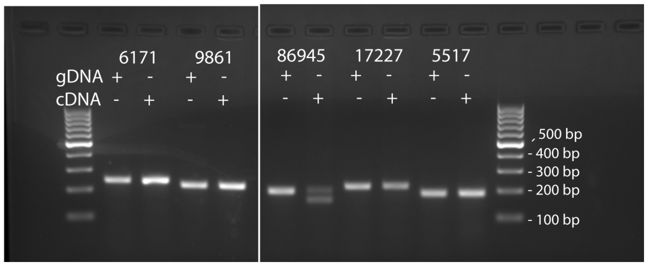

C.

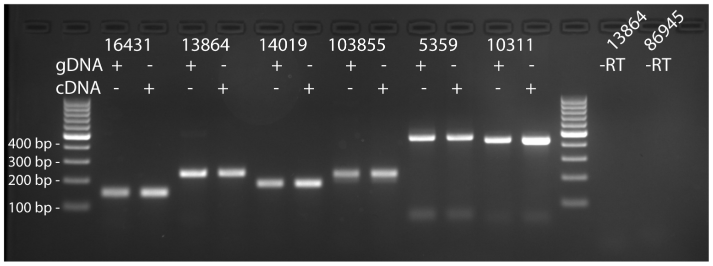

D.

```
GL50803_86945   5'-C/GTATGAT...(14 nt)..CAGCCTAACACACAG/A-3'
GL50803_27266   5'-A/CTATGTT...(13 nt)..ACAACTAACACACAG/C-3'
GL50803_15124   5'-G/GTATGTT...(10 nt)..TAACCTAACACACAG/A-3'
GL50803_17244   5'-A/GTATGTT...(87 nt)..ACAACTGACCCACAG/C-3'
GL50803_35332   5'-A/GTATGTT..(198 nt)..CCAACTGACACACAG/C-3'
GL50803_15604   5'-T/GTATGTT....(7 nt)..CCATCTAACACCCAG/C-3'
                    /.***..*                 . **.**...***/
```

E.

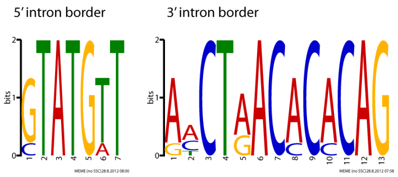

F.

Primer list : Intron verification

10311-F	CTACGAGCTCGGCGCAAACTC
10311-R	GTGCCGTGCTTTGCGATGATCTC
86945-F	GCTCCAGACCATCCGTTCCTG
86945-R	GACCCCTGCGCTGTAGCATTC
9861-F	GCATATCCAGGAGCTTCGCGAGA
9861-R	CATGGGAGCCTTTCCTTCTGAGA
16431-F	GCAAAGTTGGACATCAAGGGCTCTTC
16431-R	GCTGAATCCAGAGAGTCTTCGAAGG
13864-F	CTGGTGCCTTGTACCGCATCG
13864-R	GGAGATGTTCAGCGGAAGATCGTC
93294-F	CTCTCAACCCATCAGTGACGTATTAGC
93294-R	GAGACCTTCTGTGCCAGGACGTC
5359-F	GGACAAGCAAGCTCGCTCAAACAG
5359-R	CCTTGTCCCTGTGCTTCTCTTTATCC
14019-F	CAGCACACCTACGGATACATGGA
14019-R	CCTGCTCCTCGATGCTACAGT
103855-F	GTCAGTCATCATGGGCCTCTCA
103855-R	GAGCTGCTGACACAGCTGACG
113677-F	CTCACCGATAAAGACGCCGAGCTC
113677-R	CTCGAGCTCGGCGTCCTTCTC
5517-F	GAAGGCGAAGTACACATGCCCTTTCTGC
5517-R	CTGTGCGTCTTCACGGGCCAGA
32999-F	AGCTCAAAGAACTCAGAGAAGTGTCTC
32999-R	AGTGCGCGCTTCTAACTCTGCAG
6171-F	CGAACAATCCGTCGTATGGAGAGC
6171-R	GCTGAACATCGCATTACTCTCCTTGA
17227-F	GGTGGGCTCAAGTTTTCTACAAAGC
17227-R	GCTGCCTCACGCATGAGTAGATAG

G.

```
atgctggattctgtgatctctcttttcttgcggcccttcgcgaagaaggtgtaccagaa
M  L  D  S  V  I  S  L  F  L  A  A  L  R  E  E  G  V  P  E
         10        20        30        40        50        60
gcccaaacactcgagctgctccagaccatccgttcctggccacagatcaaggcaagcaca
A  Q  T  L  E  L  L  Q  T  I  R  S  W  P  Q  I  K  A  S  T
         70        80        90       100       110       120
tatactatcgtatgatttatttttcccaacagcctaacacacagatacagacacttaat
Y  T  I                                         I  Q  T  L  N
        130       140       150       160       170       180
aaccttgctaccagaggagtacgagcgtccaaagcgcttacagatattaccaccacattt
N  L  A  T  R  G  V  R  A  S  K  A  L  T  D  I  T  T  T  F
        190       200       210       220       230       240
ttcacttctccgcgaatgctacagcgcaggggtctttcttgtcaagacctagatgctttt
F  T  S  P  R  M  L  Q  R  R  G  L  S  C  Q  D  L  D  A  F
        250       260       270       280       290       300
catgactttagtggcgtgattgtaagaaattttattgtccatgggcatcagatccatggg
H  D  F  S  G  V  I  V  R  N  F  I  V  H  G  H  Q  I  H  G
        310       320       330       340       350       360
gttggctttactcctcttcagcttcttaga
V  G  F  T  P  L  Q  L  L  R
        370       380       390
```
